# Current Status and Future Prospects for the Assessment of Marine and Coastal Ecosystem Services: A Systematic Review

**DOI:** 10.1371/journal.pone.0067737

**Published:** 2013-07-03

**Authors:** Camino Liquete, Chiara Piroddi, Evangelia G. Drakou, Leigh Gurney, Stelios Katsanevakis, Aymen Charef, Benis Egoh

**Affiliations:** 1 Water Resources Unit, Institute for Environment and Sustainability, European Commission - Joint Research Centre, Ispra, Italy; 2 Land Resource Management Unit, Institute for Environment and Sustainability, European Commission - Joint Research Centre, Ispra, Italy; 3 Maritime Affairs Unit, Institute for the Protection and Security of the Citizen, European Commission - Joint Research Centre, Ispra, Italy; National Oceanic and Atmospheric Administration/National Marine Fisheries Service/Southwest Fisheries Science Center, United States of America

## Abstract

**Background:**

Research on ecosystem services has grown exponentially during the last decade. Most of the studies have focused on assessing and mapping terrestrial ecosystem services highlighting a knowledge gap on marine and coastal ecosystem services (MCES) and an urgent need to assess them.

**Methodology/Principal Findings:**

We reviewed and summarized existing scientific literature related to MCES with the aim of extracting and classifying indicators used to assess and map them. We found 145 papers that specifically assessed marine and coastal ecosystem services from which we extracted 476 indicators. Food provision, in particular fisheries, was the most extensively analyzed MCES while water purification and coastal protection were the most frequently studied regulating and maintenance services. Also recreation and tourism under the cultural services was relatively well assessed. We highlight knowledge gaps regarding the availability of indicators that measure the capacity, flow or benefit derived from each ecosystem service. The majority of the case studies was found in mangroves and coastal wetlands and was mainly concentrated in Europe and North America. Our systematic review highlighted the need of an improved ecosystem service classification for marine and coastal systems, which is herein proposed with definitions and links to previous classifications.

**Conclusions/Significance:**

This review summarizes the state of available information related to ecosystem services associated with marine and coastal ecosystems. The cataloging of MCES indicators and the integrated classification of MCES provided in this paper establish a background that can facilitate the planning and integration of future assessments. The final goal is to establish a consistent structure and populate it with information able to support the implementation of biodiversity conservation policies.

## Introduction

Ecosystem services are the benefits people derive from nature. Human survival and well-being depend on these services, and therefore on the conservation and the best management of ecosystems that provide them [1], [2]. Research on ecosystem services has grown exponentially during the last decade, particularly after the Millennium Ecosystem Assessment [3], [4]. According to Costanza et al. [2] and Martinez et al. [5] the oceans and especially the coastal zone contribute more than 60% of the total economic value of the biosphere. Still, data and methods to asses the provision of marine and coastal ecosystem services (**MCES**) are much more limited when compared to terrestrial assessments [6]–[9]. The few studies that deal with the assessment of marine ecosystem services have focused mainly on food production such as fisheries (e.g. [10], [11]) with other services receiving minor attention. The gap between terrestrial and marine assessments is greatest when it comes to ecosystem service mapping due to the absence or low resolution of spatially explicit information (that could be equivalent to land cover maps in the terrestrial environment) and the difficulty of quantifying ecosystem functions and processes in a highly dynamic 3D environment [12], [13]. The economic valuation of MCES is also considered an area which has lacked dedicated research efforts and within which there are still a number of challenges to be dealt with [7], [9], [14]. To add to that, existing ecosystem services classification systems have been created taking into account the terrestrial environment and in very few cases address the particularities of the marine environment [15], [16] which generates inconsistencies in the used terminologies and conceptual mismatches.

The purpose of this review was to define the status quo of ecosystem service research in marine and coastal systems. Our motivation was to establish a general background, to provide useful information for conservation policies and to identify the largest gaps to be filled by future research. To that aim, we addressed the following specific objectives:

- review the scientific literature available on the topic, analyze the coverage of that published knowledge
- extract the indicators that have been used to assess MCES, evaluate how these indicators fit within existing classifications and frameworks of ecosystem services
- analyze and highlight the main research gaps

Ideally, an ecosystem service analysis starts with the biophysical quantification and social assessment of the selected services; it leads to a valuation (monetary or other type) and, eventually, to the analysis of trade-offs, trends and scenarios [17]. Unfortunately, many ecosystem services cannot be directly quantified and, thus, researchers must rely on indicators or proxy data for their quantification. The variety of objectives and approaches of ecosystem service research and its escalating rate of publication has increased the number of proposals of ecosystem service indicators being linked to different purposes [18], [19]. Extensive reviews of such indicators, mostly focused on terrestrial systems, have been carried out by [20]–[24]. They are of great importance for the practical implementation of conservation policies and initiatives such as the Convention on Biological Diversity, the Intergovernmental Science-Policy Platform on Biodiversity and Ecosystem Services, the UN Millennium Developmental Goals, or the EU Biodiversity Strategy to 2020. Due to the relative short history of application of ecosystem services to marine environments and the difficulties mentioned above, the concept and metrics of MCES are still under development and to date no reviews of indicators of marine ecosystem services that we know of are available. The compilation of MCES indicators performed in this review could serve as an information repository, while enhancing our understanding towards ecosystem services specifically provided by the marine and coastal systems and towards key characteristics/functions that can be used for quantifying and valuing MCES.

In this paper we provide a systematic review of the scientific literature related to MCES. First we present the data structure followed in this analysis. Several classifications and analytical frameworks have been proposed to assess ecosystem services (e.g. [15], [25]–[27]). Based on our exploration of the scientific literature, we structure the results of this review around an integrated classification of MCES that addresses the specificity of the marine environment and the correspondence with other classifications. A second dimension of our data structure is the cascade model that differentiates between capacity, flow and benefit of each ecosystem service [28], [29]. Secondly, we describe and analyze the published papers and case studies under multiple perspectives (e.g. type of approach, geographical distribution, main focus). Then, we present the main outcome from this study, the extraction and classification of the indicators used to assess and map MCES. We also link, based on the existing assessments and indicators, the most studied habitats with the MCES they provide. Finally, we identify the major current knowledge gaps. In the supplementary material we synthesize other useful information for practitioners, such as the terminology used to refer to MCES in the literature or the categorization of 476 indicators compiled in this review. All the information is summarized and organized in a systematic way in order to facilitate its use by researchers and practitioners wanting to map and assess ecosystem services for marine and coastal environments.

## Methodology

### 1. Literature Search

This systematic review follows the PRISMA (Preferred Reporting Items for Systematic Reviews and Meta-Analyses) statement as a guide [30] (see table S1). The bibliographic search was performed with the SciVerse Scopus engine, arguably the largest database of peer-reviewed research literature (http://www.info.sciverse.com/). Eligibility criteria included any paper or review published between 1823 and the cutoff date 04/04/2012 with the following terms in the title, keywords or abstract: (“ecosystem service” or “environmental service”) and (“marine” or “sea” or “coastal” or “ocean”). The results summed up 986 papers that without duplicates became 563 papers. Grey literature and non-English publications are omitted from this review.

There might be publications that look at ecosystem functions or socio-economic benefits and would qualify as ecosystem services analyses, even though they do not mention “ecosystem service” or “environmental service”. However, these were not included in our search since the scope of this systematic review is to “define the status quo of ecosystem service research in marine and coastal systems” and, thus, our search was focused on papers that had framed their work explicitly in the ecosystem service concept (measuring the production and/or demand of ecosystem services). In addition, from a methodological point of view, it would be an enormous undertaking to review all the literature that refers to each MCES (from a brief bibliographic search we estimate that it could imply checking more than 50,000 publications). Still, addressing all the available thematic papers is something that could/should be done in reviews that focus on one single ecosystem service.

### 2. Selection Criteria

**Figure 1:**
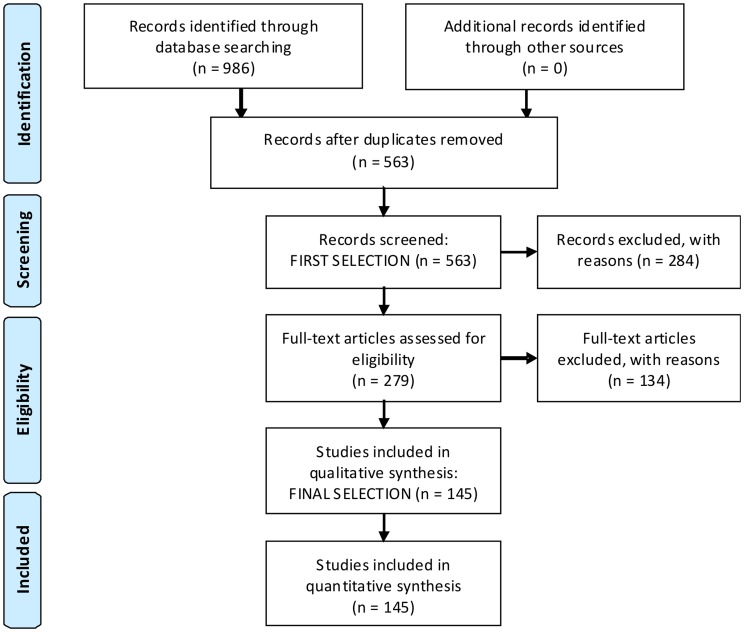
Flow diagram of the methodology and selection processes used in this systematic review. It follows the rules and templates of PRISMA (Preferred Reporting Items for Systematic Reviews [30]). The related check-list can be found in table S1.

The process of selecting papers to include in our review started with a screening of the 563 abstracts found in the previous step (fig. 1). This first selection provided a general characterization of the literature that contained marine or coastal ecosystem services in their title, keywords or abstract. The number of papers mentioning MCES increased exponentially after 2006. The occurrence of only 18 papers before 2000 indicates that the terminology and the research theme are new.

The selection criteria during the abstracts’ screening were the topic of the paper and the type of ecosystem analyzed. Articles were excluded if they were:

- Not related to MCES, total 49 papers.
- Mentioning the term ‘ecosystem service’ for justification without addressing the issue, total 122 papers.
- Based purely on terrestrial habitats, total 113 papers.

As a result, approximately 50% of the original papers were excluded at this stage. Thus, only the papers that carried out assessments of ecosystem services in marine or coastal environments, or those whose content was unclear reading the abstract alone were retained for the second step of the analysis.

The remaining 279 papers were included in the study for full text reading and further analysis (fig. 1). In this second selection process, the exclusion criteria were the same as those listed above, with one additional factor related to the language of the full text publication. Papers were left out when they:

- Were not related to MCES, total 36 papers.
- Mentioned the term ‘ecosystem service’ for justification without addressing MCES quantitatively or qualitatively, total 58 papers.
- Analyzed terrestrial habitats, total 16 papers.
- Had their full text article not available in English, 24 papers.

With this final selection, 145 studies were kept for the qualitative and quantitative synthesis (fig. 1).

### 3. Data Collection

During the final selection, information was extracted from the 145 specific studies of MCES. Data collection was organized around two main pillars: (1) the general characteristics of the paper, and (2) the parameters used in each ecosystem service assessment. In particular, the following features were extracted:

- 1a) Year of publication.
- 1b) Paper perspective: environmental, economic, social, mixed (any combination of the other options).
- 1c) Type of analysis: quantitative, mapping, qualitative, conceptual, mixed.
- 1d) Type of study area: terrestrial, marine, coastal or both.
- 1e) Number of MCES mentioned and assessed.
- 1f) Ecosystem service classification system used in the assessment (e.g. MA, TEEB, other).
- 1g) Country of institutional affiliation of the first author.
- 2a) MCES assessed, e.g. food provision, carbon sequestration (see section “Integrated classification of MCES” and correspondence table S2).
- 2b) Attainment of: quantification (in biophysical or economic terms) and/or mapping.
- 2c) Type of habitat analyzed, e.g. mangrove, estuary, open ocean (more details in section “Linking MCES assessments and habitat distribution”).
- 2d) Type of data: primary data (direct observations), simple statistics, model data (process models using indicators as variables in the equation), proxy (a single or combined indicator), expert opinion [24].
- 2e) Indicator (and unit) proposed to measure MCES (full list in table S3).
- 2f) Linkage of the indicator to the cascade model (cf. section “Conceptual data structure”): capacity, flow, benefit.
- 2g) Spatial scale of the case study: local, subnational, national, supranational, continental, global.
- 2h) Location of the study area.

Using this method, we systematically extracted from 145 articles information concerning 476 MCES indicators with their units and their corresponding case studies. The qualitative and quantitative synthesis of the indicators and case studies was used for the analysis of patterns, interpretation and gap analysis.

### 4. Conceptual Data Structure

The revision and analysis of the selected MCES studies required a comprehensive and consistent data structure that allowed fitting and comparing all the published MCES assessments and their indicators. The first dimension of this data structure was the ecosystem service classification. In order to conduct a systematic review, we integrated and harmonized the different classifications schemes used in the literature. From the selected 145 MCES papers, 68% did not follow or mention any standard classification, 15% followed the Millennium Ecosystem Assessment [25] scheme, 3% followed the proposal by Beaumont et al. [15] and the rest used other sources. This ambiguity led us to turn to the best established ecosystem service classifications, namely the Millennium Ecosystem Assessment – MA [25], The Economics of Ecosystems and Biodiversity – TEEB [26], the Common International Classification of Ecosystem Services – CICES [27] and Beaumont et al. [31]. These existing classifications either focused on terrestrial habitats and overlooked some marine aspects, or failed to accommodate all our results. Thus, we put together an adjusted classification scheme based on the combination of the previous ones while adding specificities for the marine and coastal environment. We used existing ecosystem service classes, adapted their terminology and integrated them around the main ecosystem processes, always trying to align with the empirical assessments of marine or coastal ecosystems that we found in the literature. We used this integrated classification of MCES to organize the results of this review, specifically to categorized the indicators and values provided for each service, the habitats linked to each service, and the gaps in MCES assessments.

**Figure 2:**
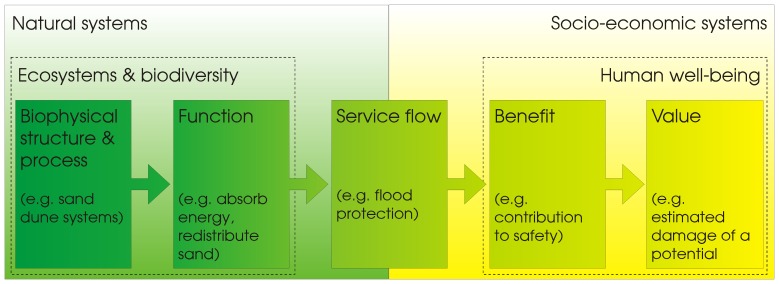
Illustration of the cascade model framed within the natural and socio-economic context. Graphic taken from [43] modified after [28], [29].

The second dimension of our data structure was a conceptual framework to analyze ecosystem services. In this review, we follow the ecosystem services cascade model, which links biodiversity and ecosystems to human well-being through the flow of ecosystem services [28], [29]. The main reason for this choice is that this model proves to be useful for framing indicators of ecosystem services with multiple perspectives, objectives and scales (e.g. [13], [32]–[34]). In the cascade model, the biophysical structure and processes of an ecosystem determine its functions, which are defined as a subset of the ecological interactions that underpin the CAPACITY of an ecosystem to provide services. Functions that ultimately contribute to human well-being are considered the FLOW of ecosystem services. The flow may be translated into specific societal BENEFIT. Different methodologies, then, allow allocating monetary or alternative values to those benefits (fig. 2). The original description of this model can be found in [28], [29] and further developments in [35]. This valuable conceptual framework allows us to structure the set of indicators and metrics found in this review into capacity or function, flow and benefit (the main steps of the cascade) (fig. 2).

## Integrated Classification of Marine and Coastal Ecosystem Services

Before entering into the results of this review, this section will present the list of ecosystem services followed in the integrated classification scheme and the reasoning behind it. This scheme is not a new classification of ecosystem services but an adaptation of the existing ones using the outcomes of this review.

**Figure 3:**
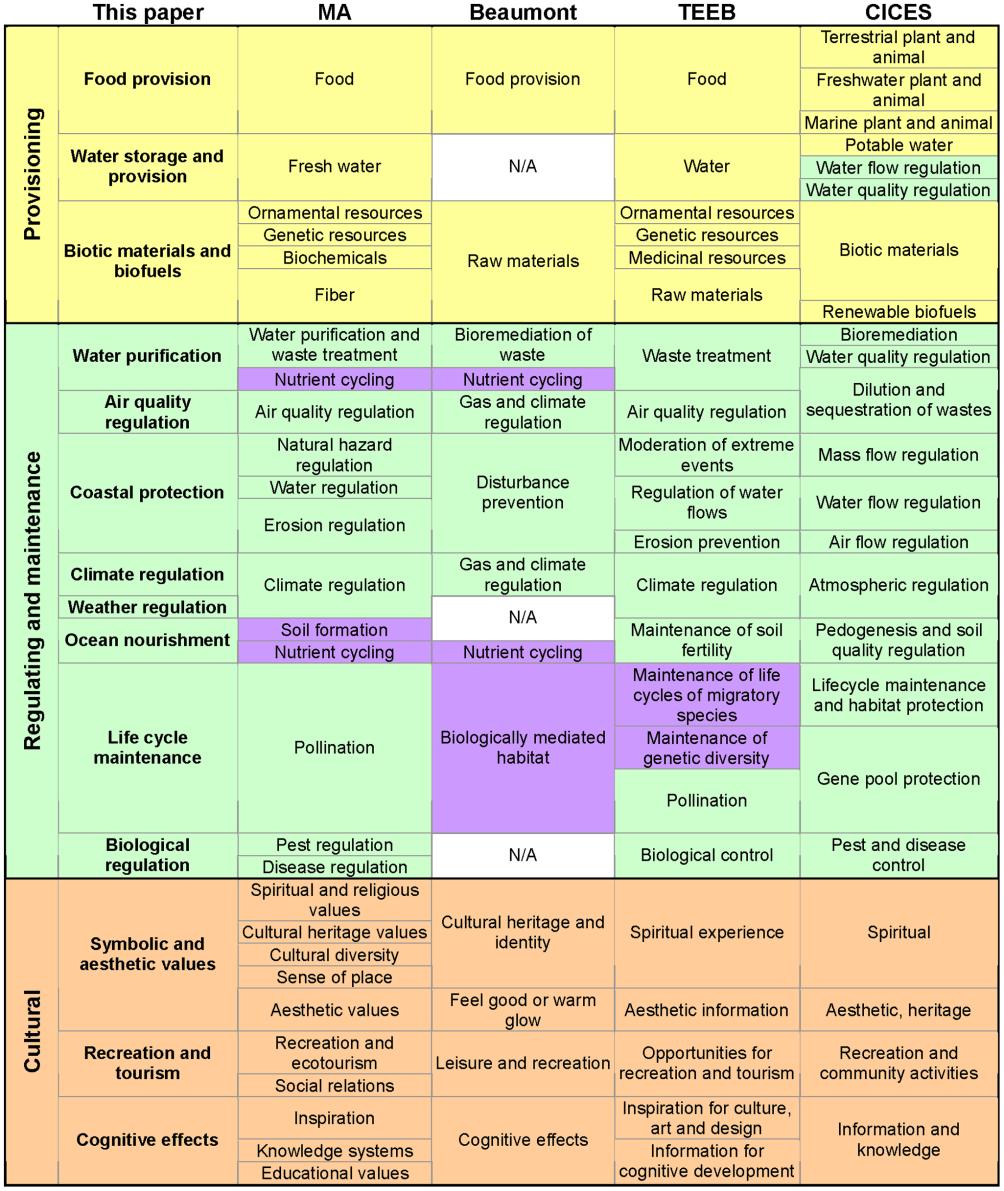
Correspondence of the integrated classification of marine and coastal ecosystem services proposed in this paper with previous classifications. The previous classifications schemes are the Millennium Ecosystem Assessment – MA [25], Beaumont et al. [15], The Economics of Ecosystems and Biodiversity – TEEB [26] and the Common International Classification of Ecosystem Services version 3– CICES [27]. N/A: not available. The colors (yellow, green, orange and purple) have been used to differentiate the categories of services (provisioning, regulating and maintenance, cultural, and supporting/habitat services respectively). MA defined three services that could not be correlated with our MCES proposal: photosynthesis, primary production, water cycling; Beaumont had an addition of two services: resilience & resistance, and future unknown & speculative benefits; and CICES v3 included abiotic materials and renewable abiotic energy, although they are no longer supported in the preliminary CICES v4.1 (available at http://cices.eu/).

The diversity of existing classifications of ecosystem services has led to difficulties and inconsistencies in comparisons between assessments [36]. However, it has been argued that a single classification scheme cannot be applicable for all habitats or assessments [37], [38] [18], [39]. This is further exacerbated for MCES due to the lack of ecosystem service studies providing sufficient examples from marine systems (e.g. [40]–[42]). An additional challenge is the matching of empirical assessments with theoretical classifications, as we faced in this review. We addressed these two issues by building an integrated and practical classification of ecosystem services tailored for coastal and marine studies (table 1). This list is the result of a critical analysis and integration of different classifications coming from MA [25], TEEB [26], CICES [27] and the marine proposal by Beaumont et al. [31] (fig. 3). The detailed description and examples provided in table 1, together with the correspondence figure 3 and the cross-reference of our list of services with the nomenclature used in the literature (table S2), allow for an easy identification and translation between different classification schemes.

**Table 1:** List and description of the integrated classification of marine and coastal ecosystem services used in this review.

	MCES	Marine/Coastal specific component	General ES definition
**Provisioning services**	Food provision	a. Fishing activities (including shellfishing) industrial or artisanal (either commercial or subsistence fishing). In general, fisheries are reported as total landings or catch per unit effort and, sometimes, corresponding jobs.b. Aquaculture is the farming of aquatic organisms, including fish, crustaceans, mollusks, seaweeds and algae.	The provision of biomass for human consumption and the conditions to grow it. It mostly relates to cropping, animal husbandry and fisheries.
	Water storage and provision	a. Water abstraction in marine and coastal environments is mostly associated to coastal lakes, deltaic aquifers or desalination plants.b. Marine water may also be used for industrial cooling processes or coastal aquaculture in ponds and raceways.	The provision of water for human consumption and for other uses.
	Biotic materials and biofuels	a. This includes medicinal (e.g. drugs, cosmetics), ornamental (e.g. corals, shells) and other commercial or industrial resources (e.g. whale oil, fishmeal, seal leather, algal or plant fertilizers).b. Biomass to produce energy can have a solid form (like wood from mangroves), liquid (like fuels extracted from algal lipids or whale oil) or biogas (from decomposing material).	The provision of biomass or biotic elements for non-food purposes.
**Regulating and maintenance services**	Water purification	Treatment of human wastes (e.g. nitrogen retention); dilution; sedimentation, trapping or sequestration (e.g. of pesticide residues or industrial pollution); bioremediation (e.g. bioaugmentation after marine oil spills); oxygenation of “dead zones”; filtration and absorption; remineralisation; decomposition.	Biochemical and physicochemical processes involved in the removal of wastes and pollutants from the aquatic environment.
	Air quality regulation	Vegetation (e.g. in mangroves), soil (e.g. in wetlands) and water bodies (e.g. open ocean), due to their physical structure and microbiological composition, absorb air pollutants like particulate matter, ozone or sulphur dioxide.	Regulation of air pollutants concentration in the lower atmosphere.
	Coastal protection	Natural defense of the coastal zone against inundation and erosion from waves, storms or sea level rise. Biogenic and geologic structures that form the coastal habitats can disrupt the water movement and, thus, stabilize sediments or create buffering protective zones.	Protection against floods, droughts, hurricanes and other extreme events. Also, erosion prevention in the coast.
	Climate regulation	The ocean acts as a sink (and only a very marginal source) for greenhouse and climate active gases. Inorganic carbon is dissolved into the seawater, organic carbon is formed through primary producers, a percentage of which is stored, and a percentage of which is sequestered.	Regulation of greenhouse and climate active gases. The most common proxies are the uptake, storage and sequestration of carbon dioxide.
	Weather regulation	For example, the influence of coastal vegetation and wetlands on air moisture and, eventually, on the saturation point and the formation of clouds.	Influence of ecosystems and habitats on the local weather conditions such as thermoregulation and relative humidity.
	Ocean nourishment	Natural cycling processes leading to the availability of nutrients in the seawater for the production of organic matter.Pedogenesis could be observed at the margin of certain wetlands and mangroves, depending on hydrodynamic conditions.	In the terrestrial realm it refers to pedogenesis and soil quality regulation.
	Life cycle maintenance	The maintenance of key habitats that act as nurseries, spawning areas or migratory routes (e.g. seagrasses, coastal wetlands, coral reefs, mangroves). These habitats and the connectivity among them are crucial for the successful life cycle of species. This also includes pollination (e.g. mangrove pollination), and seed and gamete dispersal by organisms.This service guarantees the maintenance of genetic diversity or gene pool protection.	Biological and physical support to facilitate the healthy and diverse reproduction of species.
	Biological regulation	Control of fish pathogens especially in aquaculture installations; role of cleaner fishes in coral reefs; biological control on the spread of vector borne human diseases; control of potentially invasive species.	Biological control of pests mostly linked to the protection of crops and animal production that may affect commercial activities and human health.
**Cultural services**	Symbolic and aesthetic values	Coastal communities have always shown strong bonds to the sea due to the local identity. Natural and cultural sites linked to traditions and religion are numerous in the coastal zone. Both coastal and inland societies value the existence and beauty of charismatic habitats and species such as coral reefs or marine mammals.	Exaltation of senses and emotions by landscapes, habitats or species.
	Recreation and tourism	The appeal of marine ecosystems is usually linked to wilderness, sports, or iconic landscapes and species. It can be related to coastal activities (e.g. bathing, sunbathing, snorkeling, scuba diving) and offshore activities (e.g. sailing, recreational fishing, whale watching).	Opportunities that the natural environment provide for relaxation and amusement.
	Cognitive effects	Inspiration for arts and applications (e.g. architecture designs inspired in marine shells, medical applications replicating marine organic compounds). Material for research and education (e.g. discoveries of new deep sea species). Information and awareness (e.g. respect for nature through the observation of marine wild life).	Trigger of mental processes like knowing, developing, perceiving, or being aware resulting from natural landscapes or living organisms.

MCES: marine and coastal ecosystem services. ES: ecosystem service.

Most of the cells shows 0–2 case studies, italics point to 3–6 case studies, and bold numbers refer to 7 or more case studies.

Regarding the ‘Water storage and provision’ category, the CICES classification advocates for a division between potable (drinking water) and non-potable water to feed into the economic activities and accounting tables. However, due to the lack of such data for MCES (i.e. quantification of the amount of water devoted to each use) it is not possible to make such a separation and therefore it has not been done for this classification. Similarly, we could not make the distinction between the amount of biomass used for energy (e.g. wood fuel from mangroves) and for other uses (e.g. wood for house construction) due to the lack of detailed socio-economic data in the available publications.

Abiotic raw materials and renewable abiotic energy whose availability, quantity or quality is not enhanced by living organisms or ecological processes (e.g. sand and gravel, salt, wind and wave energy) are considered as natural resources but not as ecosystem services. Water provision is a particular case. It is considered an ecosystem service since the quality and quantity of the exploitable water depends on ecological structures and processes (soil characteristics, evapotranspiration, denitrification, microbial activity, etc.).

‘Coastal protection’ is a combination of the so-called services ‘hazard prevention’ or ‘flow regulation’ and (soil) ‘erosion prevention’. In marine systems, all these processes act over a narrow coastal strip, and both the causes (e.g. waves, storm surge) and protection against them (e.g. resistant geomorphology, presence of biotic structures) are similar for hazard and erosion prevention [43]. For this reason, in marine and coastal environments, these have been grouped together in the classification system.

Previous classification systems traditionally incorporate weather regulation as part of climate regulation. Here the two services were considered separately based on the differences in scale, processes and beneficiaries. Weather regulation refers to meteorological processes acting at local scale and affecting only local residents. Most of the processes are related to the water cycle. Climate regulation entails global climatic processes affecting in the long-term the global atmospheric composition. Most of the processes are linked to the carbon cycle.

Ocean nourishment is proposed as the marine counterpart of terrestrial soil formation, structure and quality. Similarly to the support soil provides for agriculture, nutrient rich sea water maintains fish provisioning and includes the ecosystem service of nutrient cycling.

By adopting the CICES general structure, our integrated MCES classification can be directly linked with the framework of the UN System of Environmental-Economic Accounts (SEEA) and with several standard product and activity classifications, namely the International Standard Industrial Classification of All Economic Activities, the Central Products Classification, and the Classification of Individual Consumption by Purpose which we feel will be relevant for future progression of MCES work. In addition, our classification is closely linked to the TEEB proposal [26] (based on previous research by de Groot et al. [40]), which captures the main ecological processes and ecosystem services principles. We also observed the prioritization of services and specific marine nomenclature used by Beaumont et al. [31].

## Review of Marine and Coastal Ecosystem Services

### 1. Analysis of Published Papers and Case Studies

**Figure 4:**
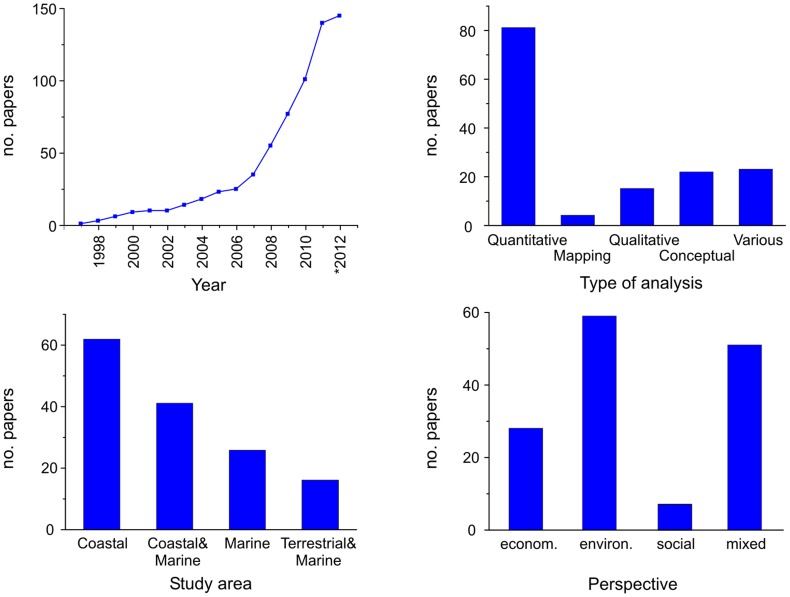
Data and analysis from the selected 145 MCES assessments. A: Number of publications per year. *The year 2012 covers from January 1^st^ until the cutoff date April 4^th^. B: Number of studies per type of analysis. C: Number of papers per type of environment analyzed. D: Number of publications per scientific discipline.

The number of papers assessing MCES increased exponentially after 2006 (fig. 4A), with only 10 articles published before 2000. From 1997 to 2006 the average rate of publication was 2.5 papers per year. Thereafter the publication rate rose to 23 papers per year. Quantification was the main type of analysis (56%) followed by mixed analyses (16%), conceptual frameworks (15%) and qualitative assessments (10%). Only a few studies actually produced maps of ecosystem services (3%) (fig. 4B). For the 145 selected papers, the study area most frequently analyzed was the coastal zone (43%) or the coastal and marine areas together (28%). The open sea was the focus of 18% of the articles while both terrestrial and marine environments (in a broader sense) were dealt with in 11% of the cases (fig. 4C). Most of the selected articles (41%) had a biophysical or environmental perspective, over a third of them (35%) chose a multidisciplinary approach (mostly environmental-economic), one fifth (19%) were economic valuation studies and the remaining (5%) were social studies (fig. 4D). Finally, of the number of ecosystem services assessed in each paper, half of the articles (48%) studied just one service, 39% analyzed between 2 and 5 services, and the remaining papers (13%) assessed 6 or more services.

**Figure 5:**
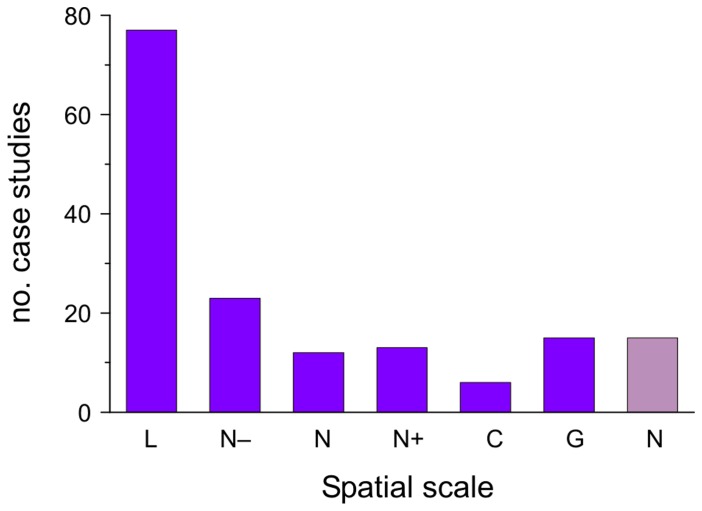
Spatial scale of the MCES case studies found in the literature. L: local, N-: subnational, N: national, N+: supranational, C: continental, G: global, N: no case study.

**Figure 6:**
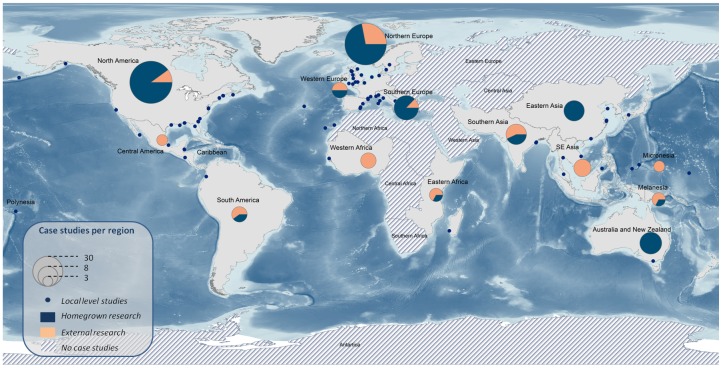
Map illustrating the location of the MCES case studies and the affiliation of the first authors. The pie size represents the number of studies carried out per region. The regions definition follows the Global Administrative Units and Layers scheme (http://www.fao.org/geonetwork/srv/en/metadata.show?id=12691). Points depict the location of the local level case studies. The colors within the pie show the percentage of studies carried out by researchers from the same region (blue), or from different regions (orange). In regions such as Central and South Africa, or West and Central Asia no MCES assessments have been found.

The selected MCES papers summed up 161 case studies, meaning that only a few (e.g. [44], [45]) of the 145 papers analyzed more than one case study. Of those case studies, 48% were carried out at a local scale, followed by 14% at subnational level, 7% at national scale, 8% at supranational level, 4% at continental scale and 9% at global scale (fig. 5). The remaining 15 articles were either reviews, MCES modeling assessments, or were purely conceptual and had no case study. The location of the local scale case studies is shown in figure 6.

One third of the case studies were located in Europe and nearly one fifth (18%) in North America. Asia accounted for 13% of the case studies, while Australia, New Zealand and the Pacific Islands represented 8%. Africa and Central and South America amounted to 4 and 5% of the case studies respectively (fig. 6). The remainder were either global or had no case studies (see above). Half of the continental scale studies and half of the national level studies (i.e. the broader assessments) were located in Europe.

We also investigated the correspondence between the affiliation of the first author and the case study. Most of the selected papers were led by USA or UK institutions (49 and 25 articles respectively). Researchers from Australia, Sweden, Spain, China and Germany produced between 6 and 12 MCES papers each. The remaining articles were produced in other 17 countries, of which 6 are EU Member States. It is remarkable that the location of one third of the case studies corresponded to the country of the first author’s hosting institution, while for two thirds of the cases (notably in SE Asia, Western Africa and Central America) this is not the case (fig. 6). If the assumption is that the research funding is originating from the same country as the affiliation of the first author, then most of the support/funding for MCES studies come from USA, UK and other EU-15 countries. It is remarkable that in Western Africa and South-East Asia the recorded assessments have been have been carried out by external researchers.

The 145 papers analyzed propose 476 MCES indicators that are listed and classified following the cascade model in table S3. This gives a mean value of more than three indicators per paper. On average, there are 47 indicators for each provisioning service (summing up 141 indicators in total), 27 indicators for each regulating and maintenance service (up to 214), and 27 indicators for each cultural service (up to 80) (table 2). Following the cascade scheme, 224 of the MCES indicators are linked to benefit, 111 to flow and 141 to capacity. There is a group of 41 indicators that are not affiliated to a specific service but attempt to quantify or describe the total capacity or benefit from all MCES.

**Table 2:** Quantitative synthesis of the results shown in table S3, which compiles the MCES indicators found in this literature review.

	No. indicators		
MCES	Capacity	Flow	Benefit
Food provision	25	27	51
Water	*0*	*3*	*4*
Biotic materials and biofuels	*2*	10	19
Water purification	22	20	12
Air quality regulation	*0*	*1*	*0*
Coastal protection	16	7	30
Climate regulation	14	10	10
Weather regulation	*0*	*0*	*0*
Ocean nourishment	11	*4*	*3*
Life cycle maintenance	27	8	15
Biological regulation	*0*	*3*	*1*
Symbolic and aesthetic values	*0*	*4*	12
Recreation and tourism	*5*	11	36
Cognitive effects	*1*	*3*	8
*All MCES together*	18	*0*	23

The division into capacity, flow and benefit comes from the application of the cascade model (see section “Conceptual data structure”). The lowest values in this table are written in italics.

As already mentioned in the section “Literature search”, this review considers only studies that explicitly frame their research in the ecosystem service concept, i.e. they attempt to capture and demonstrate the link between ecological processes and benefits for society. Hence, there are many marine and coastal studies in the literature that propose indicators that can provide useful metrics for ecosystem assessments but are not covered in this review. It is out of the scope of this paper to summarize all the available biophysical and socio-economic indicators in the marine realm. However, the categorization of MCES indicators provided in this review may provide a route for the preceding work on natural resources, biophysical processes, environmental economics or social studies (not covered in the review) to be reconsidered, re-analyzed and re-launched in an ecosystem service context.

### 2. Present Focus of the MCES Assessments

The analysis of MCES is a new field of research with relatively small presence in the literature before 2006. This is a common trend in ecosystem service research, whose most specific journals (*International Journal of Biodiversity Science, Ecosystem Services & Management* and *Ecosystem Services*) were established in 2010 and 2012 respectively. Many authors consider that this ‘explosion of interest’ was partly generated by the Millennium Ecosystem Assessment [3], [4]. The exponential publishing rate is enhancing our knowledge of MCES, but the lack of established conceptual frameworks, indicators and metrics complicates the integration of data and information. In addition, MCES is currently a fashionable concept commonly mentioned in the justification of studies but less commonly applied and assessed, as demonstrated by the content-based elimination of 75% of the original papers for this review (fig. 1). This further complicates knowledge and data mining.

The analysis of MCES requires an interdisciplinary approach that is followed at least in 35% of the studies. Most of the publishing efforts come from environmental fields. Social sciences, crucial not only for the analysis of cultural services but also for most of the valuation methodologies and the analysis of beneficiaries, are clearly underrepresented in MCES. These main disciplines affect the compartments of the cascade scheme addressed by each paper, with environmental studies usually focused on capacity (e.g. [46], [47]) and economical studies on benefit (e.g. [48], [49]). The comparison and eventual combination of these different methodologies lies in the use of a common conceptual framework that classifies each service and indicator, such as is proposed in this paper.

In terms of the type of MCES assessments found, more than half of the reviewed papers provide quantitative indicators or measures. Qualitative assessments (one tenth of the papers) collected mostly expert opinion or preferences based on questionnaires addressed to specific stakeholders or lay citizens. Apart from the mixed analyses that may contained some geographical data (e.g. [50]), only four MCES papers were identified as mapping approaches and all of them focused on the coastal zone: Costanza et al. 2008 [51] mapped and valued storm protection by wetlands at a national scale; Edwards et al. [52] mapped spatial dependencies of ecological processes for life cycle assessment and fisheries support; Feagin et al. [53] mapped the distribution of salt marshes plants and the value of five MCES under sea level rise scenarios at high resolution; and Ruiz-Frau et al. [54] roughly mapped stakeholders’ values for 14 different types of societal benefits derived from the marine environment. Due to the absence of spatially explicit information about ecosystem services, in most of the cases only coarse estimates or statistics at national level have been used (e.g.[55]–[57]) usually with uncertainty in the location of supply and demand. Some examples of modeling tools to map MCES were provided by the InVEST initiative – Integrated Valuation of Environmental Services and Tradeoffs ([58], [59]).

Constraints for developing MCES mapping exercises include (1) the lack of coverage and resolution in the available natural and socio-economic data (e.g. habitat mapping), (2) the ambiguity of maritime boundaries or eventual assessment units, (3) the multi-dimensional structure formed by benthic and pelagic habitats, more dynamic and less explored than terrestrial ecosystems, and (4) the difficulty to assess connectivity between habitats ([12]). The spread and development of geographically explicit MCES assessments would foster and inform not only biodiversity conservation policies [13] but also the application of ecosystem-based marine spatial management where all societal interests and natural benefits could be represented [60].

## Indicators of Marine and Coastal Ecosystem Services

The cascade model provides a useful framework to contextualize the links between the natural provision of ecosystem services, the effects on human well-being, and the way the institutional and social responses may influence the state of the ecosystems and, therefore, their potential to provide further services [28], [29], [35]. Using such a framework allows the ecosystem service information to be translated into appropriate institutional and social responses [61], and highlights where future research should be focused to fill the major gaps.

The analysis of MCES indicators in the cascade model (table S3 which is summarized in table 2) provides a useful overview of what elements of the model received the greatest attention. For both provisioning and cultural services the number of indicators for benefit is high, while those that measure capacity and flow are lacking. Some provisioning and cultural services like water or symbolic and aesthetic values have no capacity assessments. On the other hand, for regulation and maintenance MCES, the number of indicators measuring the capacity is high and those which address the benefit are lacking, with the only exception of coastal protection. Ideally all parts of the model should ultimately be well represented.

Food provision, in particular fisheries, is the most analyzed MCES, probably due to its economic relevance and the existence of market prices to value it, notwithstanding that ecosystem valuations should not confuse prices with values [62]. Some of the most meaningful indicators of this service include (table S3):

- Capacity: abundance or biomass of commercial marine living resources, fish diversity, food web structure, sea food quality. It would be particularly relevant to analyze the evolution of this capacity through time and to develop scenarios.
- Flow: catches (preferably with spatial distribution), landings, number of viable fisheries.
- Benefit: income from fisheries, jobs, community dependence and perception on fisheries. The value of fish, commonly used as an indicator, should take into account not only market values but also subsidies. Alternative non-monetary values could be related to human diet quality.

Water purification is the most frequently studied of the regulating and maintenance services. Indicators related to water purification mainly focus on the presence of excess nutrients (eutrophication) or suspended particulate matter [63], [64], with few examples of other pollutants [65]. The benefit part of the assessments is characterized by a large variety of valuation techniques and well-being aspects (table S3), ranging from replacement costs for different water treatments to the promotion of various uses like coastal recreation.

Coastal protection is the third most analyzed MCES, accounting for 11% of the indicators in table S3. The indicators found in the literature review are in line with the definitions of table 1, even if some studies put more emphasis in hazards and inundation [66], [67] and others on erosion [68], [69]. Local case studies in particular provide a detailed insight of the processes and values involved [70], [71]. The quantification of the protection capacity, flow and benefit is proposed by [43]. In general, coastal protection indicators refer to the presence of biotic structures that disrupt water movement, coastal exposure, public awareness, and avoided or replacement costs (table S3).

Recreation and tourism is the next most commonly assessed MCES. The list of available indicators covers many relevant aspects of this service especially on the benefit side, e.g. estimated economic value of/income from tourism and recreation, perceived benefit from recreational activities or for the presence of a marine protected area, visitors and travel cost, willingness-to-pay to enjoy a natural area (table S3). The list is deficient in capacity indicators, where we suggest taking into account the naturalness of the ecosystem (for example, using an analogous to the terrestrial hemeroby concept [72], [73]) and the accessibility of the site. The state of the ecosystem, in particular related to pollution and other disturbing factors, is already covered by some of the proposed indicators [74], but could be explored further.

A substantial number of studies proposing 10% of the indicators in table S3 refer to life cycle maintenance. The objective of these studies is highly variable due to the broad topic of this service and the complexity of processes, species and habitats involved. In most of the cases it has been interpreted as a fishing support service [75], [76], which represents a small portion of the role of this crucial service to maintain all ecosystems. The differentiation between indicators for fisheries and indicators for life cycle maintenance in table S3 is based on the original authors’ classification, but in our opinion some of them could be interchanged. In addition, valuing this complex ecological service through willingness-to-pay or other stated preferences’ techniques that do not necessarily correlate with benefit or utility [62] can be misleading.

Climate regulation is most often addressed as an ecosystem service through the carbon cycle, with nearly no reference to nitrogen climate active gases. We differentiate here between the exchange of inorganic carbon which occurs at the air-sea interface and the generation of organic carbon through primary production. Ultimately, in terms of an ecosystem service, the value of the uptake of carbon by the ocean is effective only when it is stored for extended periods (years) or sequestered from contact with the atmosphere. In oceanic waters, phytoplankton communities are responsible for the uptake of inorganic carbon through photosynthesis, yielding a global annual net primary production equivalent to that of terrestrial systems [77]. The fate of the production of organic carbon determines whether such processes ultimately contribute to climate regulation. Most of the production generated by phytoplankton photosynthesis in open water habitats is recycled within the system with only a small percentage ultimately being sequestered in deeper layers and bottom sediments through sinking particles (biological pump) [78]. In coastal areas, specific habitats, for instance mangroves or seagrass beds, are important for carbon storage making considerable contributions to the global carbon stocks despite their limited geographical range and extent [79], [80]. Indicators tracking the state and temporal trends of such habitats provide valuable information for the assessment of this MCES. In the context of the results found in this review (table S3), indicators of the capacity of the system include all measures of stock and concentration; flow is monitored through uptake, accumulation or sequestration rates; and benefit is usually estimated with the market value of carbon. However, some indicators convert the uptake of carbon (primary production) to a monetary value without consideration for the fate of the carbon fixed, even if most of this carbon is not stored or sequestered [78].

Biotic materials and biofuels are poorly assessed. The indicators provided refer to a few materials (mostly mangrove wood) and many of them are based on experts’ or stakeholders’ opinion instead of data on production or extraction (table S3).

The least studied MCES are weather regulation, air quality regulation, biological regulation and water provision. Also, the cultural MCES are relatively poorly assessed and, especially, quantified. Certain compartments of the cascade scheme are especially difficult to fill in with the available MCES indicators (italics in table 2). In particular all columns of the four least studied MCES above as well as the capacity of biotic materials, the flow and benefit of ocean nourishment, and the capacity and flow of both symbolic and aesthetic values and cognitive effects are limited.

The indicators that pull all MCES together can describe very relevant natural or socio-economic characteristics, like local extinctions of species or social perception of all the ecosystem services (table S3), but they are difficult to classify and use in MCES analysis. These indicators can play an important role for conservation and communication purposes but we consider that their applicability for future MCES assessments may be limited.

A lot of valuation studies use the benefit transfer technique to estimate the value of MCES (e.g. [16], [81], [82]). In this review we found that most of these studies used the values provided by [2]. This pioneering work triggered the discussion on the value of nature and conservation in scientific and policy fora, fostering the development of ecosystem service approaches. However, we have some concerns on the use of global average values for specific case studies (e.g. [56], [83]), especially noting that data sources in marine biomes were very limited in Costanza et al. [2] (e.g., only 6 services with one valuation study per service were considered for the open ocean, and only 2 services with one valuation study each referred to seagrasses and algal beds). Progress should be made in developing these techniques for comprehensive valuations [84].

## Linking MCES Assessments and Habitat Distribution

The size and conservation status of natural habitats have direct implications on the provision of ecosystem services [85]. This provision may be dominated by certain species or trophic levels thus showing strong links with the distribution and health of particular habitats [86]. For a detailed correlation of 56 marine biotopes with the goods and services they may provide, see Salomidi et al. [87].

Most of the case studies analyzed in this review carry out their assessments in a few habitats or environments (table 3), sometimes due to their particular relevance in the provision of a service, but also for other practical reasons like the expertise of the authors or the accessibility of the study area. For many relevant habitat/service combinations, e.g. oyster reefs/food provision, seagrass meadows/coastal protection, seagrass meadows/biotic materials, macroalgal beds/life cycle maintenance [87], none or too few MCES case studies have been published, which reflects a poor understanding of the system and, thus, a poor support for any biodiversity policy. The development of further MCES assessments (either qualitative or quantitative) should be especially promoted in those cases. There are many more indicators relative to these habitats that could be used as proxies for ecosystem services even if they were not framed in the ecosystem service concept. However, as already mentioned in sections 2.1 and 4.1, it is not in the scope of this review to cover all the available thematic papers. We are only reporting on indicators that have been used explicitly in the framework of MCES. Nevertheless, the analysis shown in this section may open the door for previous studies, not framed in the ecosystem service concept, to be adapted and re-launched under this topic, to fill the knowledge and methodological gaps, and to inform biodiversity conservation policies.

**Table 3:** Matrix showing the published assessments of marine and coastal ecosystem services (MCES) and the habitats where they were measured.

	General				Habitat specific												
MCES	coastal zone	coastal and marine	continental shelf	open ocean	beach	dune	coastal wetland	man-made structure	estuary	mangrove	coral reef	maerl bed	oyster reef	macroalgal bed (incl. kelp)	seagrass meadow	hard substrata	unconsolidated sediment
Food provision	*3*	**7**		**17**		1	*6*	1		**20**	**12**			*3*	*4*	1	2
Water storage and provision			1	1	2	2	2		1								
Biotic materials and biofuels		1		*4*		1	1		1	**11**	*3*						
Water purification	**7**	*3*		*4*	1	1	**8**	1	2	*3*	2		2		1		2
Air quality regulation						1											
Coastal protection	*5*	1		1	2	*3*	**15**		*3*	**15**	*4*			1	*3*		
Climate regulation	2	1		*5*		1	*6*		1	**10**				2	*3*		2
Weather regulation																	
Ocean nourishment		*3*	1	2		1	2			2	1				1		2
Life cycle maintenance	*3*	2	1	**7**	*4*	2	**7**	2	2	**7**	*5*	1	1	2	*4*	1	1
Biological regulation		1															
Symbolic and aesthetic values	2	1	1	2	2	2	2	1	1	1	2						
Recreation and tourism	1	1			*5*		*6*										
Cognitive effects		1		*3*		1	1			2	2						
ALL MCES	2	*4*		*6*			2		1	2	1				1		

Most of the MCES case studies are found in mangroves and coastal wetlands, which form key habitats for the functioning of marine ecosystems and the provision of MCES. Nonetheless, these habitats are being lost or converted at alarming rates, i.e. 35% of mangroves in the last few decades or 20% of coastal wetlands annually in some places [25], [88]. These two reasons probably make the case for many researchers to focus their assessments in mangroves and coastal wetlands with the final goal of contributing to their conservation. Provisioning services in mangroves and some regulating and maintenance services both in mangroves and coastal wetlands (namely water purification, coastal protection, climate regulation and life cycle maintenance) are the most commonly assessed.

Coral reefs are the third best studied habitat, mostly because they provide habitat, spawning and nursery grounds for economically important commercial fish species [89], which is analyzed as food provision or life cycle maintenance services. Coral reefs also provide coastal protection from storms and erosion [89]–[91]. Seagrass meadows have been studied mostly due to their role in nursery, carbon storage and erosion control [68], [92]. Beach and dune systems are also the focus of several case studies that, contrary to what could be expected, do not concentrate only in coastal protection and recreation and tourism [82], [93], [94]. The rest of the habitats in table 3 show a marginal number of case studies, while for many other habitats (e.g. mussel beds, seamounts, oceanic ridges, hydrothermal vents, marine caves) no MCES assessment was found in our review. These gaps have to be filled by future research otherwise the value and distribution of services provided by marine ecosystems will be greatly underestimated.

Another important gap is that of the offshore ecosystems (the ‘open ocean’), currently the focus of only 15% of the case studies and mostly related to fisheries. The deep sea, but also the water column and benthic environment beyond the shelf edge, are the largest unknown under the ecosystem service perspective.

One third of the case studies refer to broad geographic areas, like the ‘coastal zone’ (with a terrestrial sense) or ‘coastal and marine’ in general. This ambiguity is not unusual in ecosystem service assessments that do not follow a mapping approach [13] and is even more understandable when dealing with marine assessments, where habitats’ distribution and other marine delimitations are usually not available or are under discussion [95], [96]. The lack of precise delimitations in most of the MCES case studies is due to the lack of disaggregated data. To date several habitat mapping efforts have been carried out globally at different spatial and temporal resolutions [97], [98]. Still, there are large regions where the spatial distribution of all relevant marine ecosystem components is unknown, which hampers the mapping and assessment of many MCES. Hence, to reliably map and assess the state of ecosystems and their services, as demanded by global and regional policies, further effort and funds should be devoted to ecological mapping, especially in data-poor regions.

## Gaps and Recommendations

This review summarizes the status quo of ecosystem services associated with marine and coastal environments. The harmonized data structure proposed in this paper and the cataloging of MCES indicators aim to facilitate the planning and integration of future assessments by showing where and what metrics have been used and can be used. This can be particularly relevant for the implementation of certain conservation policies that required assessing ecosystem services at national level in 2014 for the EU Biodiversity Strategy or 2015 for the Intergovernmental Science-Policy Platform on Biodiversity and Ecosystem Services. For example, the implementation of Target 2 Action 5 of the EU Biodiversity Strategy is currently attempting to develop several pilot case studies, of which one will be related to marine ecosystem services. The required mapping and assessment of ecosystems and their services is thought to be based on indicators, and there is still no agreement on the number or type of indicators/metrics.

The following list highlights the main gaps and recommendations extracted from this review:

- Although a wealth of literature refers to ecosystem services on marine and coastal environments, 75% do not relate to assessments and the remaining 25% are biased towards commercial fisheries and their maintenance. The best known classifications and reviews of ecosystem services miss a fair representation of marine and coastal examples.
- Social sciences and mapping approaches are clearly underrepresented in MCES. In many marine regions the absence of the necessary information on the spatial distribution of ecological components remains a bottleneck that might prevent future progress on mapping MCES.
- Most of the MCES case studies concentrate around Europe and North America, while large regions of the world have no published assessments. USA and UK institutions are the most prolific in this field.
- Most of the MCES assessments deal with coastal habitats, while the area beyond the shelf edge represents less than one fifth of them. Most of the positioned case studies are located in mangroves (for provisioning and regulating and maintenance services) and coastal wetlands (for regulating and maintenance services). The deep sea and particular benthic habitats are mostly lacking in MCES assessments.
- The main gaps in MCES indicators are related to capacity for provisioning and cultural services, benefit for regulating and maintenance services, and service flow in all the categories. The average number of indicators available for provisioning services surpasses that of regulating and maintenance or cultural services.
- The most commonly studied MCES are: food provision (fisheries), usually using market values; water purification, practically focused on nutrients and suspended matter; coastal protection, with an assortment of relevant indicators; recreation and tourism, relatively deficient in capacity indicators; life cycle maintenance, mostly interpreted as the fishing support service and, thus, only partially assessed; and climate regulation, usually disregarding the timescale and nature (organic/inorganic) of the processes involved. The remaining services are largely overlooked in marine and coastal environments.

## Supporting Information

Table S1 **PRISMA checklist [1] applied to this review paper.**
(DOC)

Table S2 **Compilation of terms used in the literature to refer to each marine and coastal ecosystem service (MCES).** No examples (and, therefore, no synonyms) were found for weather regulation.
(DOC)

Table S3 **List of indicators and units found during the systematic review of published marine and coastal ecosystem service (MCES) assessments.** Indicators are classified following the cascade model into capacity, flow and benefit (see section 2.4 and fig. 2).
(DOC)
