# Supplementary material for: Current Status and Future Prospects for the Assessment of Marine and Coastal Ecosystem Services: A Systematic Review
Source: PLoS One. 2013 Jul 3;8(7):e67737. doi: 10.1371/journal.pone.0067737 (PMC3701056; doi:10.1371/journal.pone.0067737)
Supplement: Table S2 — Compilation of terms used in the literature to refer to each marine and coastal ecosystem service (MCES). No examples (and, therefore, no synonyms) were found for weather regulation. (DOC) [file pone.0067737.s002.doc]

**Table S2**

| **MCES** | **Labels for ecosystem services found in this literature review** |
| --- | --- |
| Food provision | agroecosystem productivity; aquaculture potential; availability for harvest; biosecurity; captured fish; coastal artisanal fisheries; coastal fisheries; commercial fishing; consumptive use of a fish species; employment as fishermen; fish catch; fish consumption; fish food; fish growth; fish production from aquaculture; fish production from fisheries; fish sales; fish take; fisheries; fisheries support; fishery; fishery goods; fishery products; fishery support; fishing; fishing and fishing related activities; food; food from fisheries; food production; food provision; food resources; habitat services; harvest; mangrove-fishery linkages; off-shore fisheries; prawn, mollusk and crab production; provisioning; provisioning of protein and other products; quality of food provided to first-order consumers; quantity of food provision to first-order consumers and O2/CO2 exchange; seafood; seafood consumption; seafood provision; shrimp and fish; shrimp and fish harvesting; shrimp revenues; spawning stock production; supporting fish communities; supporting offshore fisheries |
| Water storage and provision | drinking water; fresh water; irrigation; water supply |
| Biotic materials and biofuels | biomass production; building resources; clearance logging; construction materials; coral mining; direct extractive use; energy provision; fiber and fuel; firewood and building materials; fuelwood; timber; construction material for houses, furniture, agricultural equipment, fishing boats, fishing equipment, fences, small poles, and wood for bridges; genetic resources; mangrove harvesting; mangrove resources; marine aggregates; materials; medicinal resources; mineral extraction; natural products; pharmaceutical products; raw materials; seaweed farming; wood; wood provision |
| Water purification | biodiversity; biofilter function; bioremediation; bioremediation of waste; buffering the nutrient runoff; cycling of nutrients; denitrification, mineralisation; efficient energy transfer to higher trophic levels ; filtering and detoxification; mitigation of eutrophication; nitrogen recycle/cycling; nitrogen removal; nitrogen, phosphorus and heavy metals retention; nutrient abatement; nutrient cycling; nutrient reduction; nutrient regulation; nutrient retention; oxygen production; reduced marine eutrophication effects; seawater quality improvement; toilet function; waste assimilation of nutrients and carbon dioxide; waste treatment; wastewater treatment; water and substratum quality; water clarity; water purification; water quality; water quality maintenance |
| Air quality regulation | air quality regulation |
| Coastal protection | coastal defence; coastal property protection; coastal protection; coastal protection of hurricanes; coastal sea defence; coastline stabilisation; dissipate wave energy; disturbance prevention and alleviation; disturbance regulation; environmental disturbance prevention; flood control; hurricanes protection; land building/retention; natural barriers to storm events; natural hazard regulation; protection from erosion and sand blasting; protection of shoreline against erosion and flooding; reduction of coast erosion; reef protection; sediment accumulation; sediment deposition; shoreline buffering; shoreline erosion control; shoreline protection; shoreline stabilization; soil retention; storm protection; storm surge protection; storm, flood and erosion control; water recycling; water regulation; water storage; wave and wind attenuation; wave attenuation |
| Climate regulation | biogeochemical services; biomass production; carbon; carbon and nitrogen sequestration; carbon and nitrogen storage; carbon cycling; carbon dioxide regulation; carbon flow; carbon sequestration; carbon stock; carbon storage; climate regulation; fixation of carbon dioxide; gas and climate regulation; gas exchange and carbon storage; nitrogen retention; primary productivity; productivity; sequestration of atmospheric carbon dioxide |
| Ocean nourishment | cycling of nutrients; food availability; food web; linking services; mangrove nutrient content; mediation of nutrient exchange; nutrient cycling; nutrient regeneration; nutrient regulation; nutrient stocks; nutrients concentration; nutrient uptake; photosynthesis; primary production; soil formation; trophic transfer |
| Life cycle maintenance | biodiversity; breeding and feeding ground; conservation designations; coral reef conservation; diversity; ecosystem health; fish nurseries and productivity effects; fish nursery habitat; habitat; habitat function; habitat provision; habitat structure used for settlement and as nursery ground; life support; life-support input; living space for introduced and migratory species; nursery; nursery habitat; nursery habitat for offshore fisheries; nursery services; pollination; primary productivity; provision of habitat; provision of nursery habitat; quality as nursery, feeding and breeding habitat for fishery species of commercial and recreational value; refuge; refugia; regulate trophic structure; shelter for fauna; nursery and refuge for fauna; structure of the benthic community |
| Biological regulation | biological control; disease regulation; pest regulation |
| Symbolic and aesthetic values | aesthetic; aesthetic beauty and intellectual stimulation; aesthetic value; aesthetic view; cultural; cultural and spiritual; cultural heritage; cultural values; heritage; identity; inspiration of art, folklore and architecture; landscape suitability for industrial use; place identity; seascape perception; social relations; spiritual and historic |
| Recreation and tourism | aesthetic and recreation; aesthetic and recreational values; aesthetic attraction; amenity from coastal defence; amenity, recreation and biodiversity; bird shooting and wildfowling; coastal recreational benefit; cultural; ecotourism; ecotourism and environmental education; hunting and bird watching tourism; industry and tourism; leisure; leisure and recreation; marine leisure and recreation industry; non-consumptive use of popular observable species; recreation; recreation and cultural; recreation and tourism; recreation service; recreational; recreational and aesthetic; recreational appeal; recreational benefits; recreational fishing; recreational use of coastal areas; recreational uses; scuba; tourism; tourism and local recreation; tourism attraction; tourism opportunities; tourism potential; wildlife viewing |
| Cognitive effects | cognitive value; cultural; cultural and education; educational resource; field laboratory; information services; marine biodiversity conservation; materials for kastom art and education; research; research service |

Compilation of terms used in the literature to refer to each marine and coastal ecosystem service (MCES). No examples (and, therefore, no synonyms) were found for weather regulation.
