# Supplementary material for: Current Status and Future Prospects for the Assessment of Marine and Coastal Ecosystem Services: A Systematic Review
Source: PLoS One. 2013 Jul 3;8(7):e67737. doi: 10.1371/journal.pone.0067737 (PMC3701056; doi:10.1371/journal.pone.0067737)
Supplement: Table S3 — List of indicators and units found during the systematic review of published marine and coastal ecosystem service (MCES) assessments. Indicators are classified following the cascade model into capacity, flow and benefit (see section 2.4 and fig. 2). (DOC) [file pone.0067737.s003.doc]

**Table S3**

| **MCES** | **MCES indicators found in this review under the cascade scheme** | | |
| --- | --- | --- | --- |
|  | **Capacity** | **Flow** | **Benefit** |
| **Food provision** | Relative fish abundance based on catch per unit effort (CPUE) [1] | Artisanal fishery catch [2] | Fish sales (USD/yr) [3] |
|  | Density of fish (weighting factor) [4] | Shrimp landings (t/yr) [5] | Economic benefits from fisheries (USD/yr or USD/km^2^) [6] |
|  | Coral size, substrate cover, fish diversity and biomass [7] | Harvested mussels (n°) [8] | Value of the average daily catch (USD/ha) [9] |
|  | Fish abundance per site [10] | Fish catch (kg/yr) [3] | Financial income from fisheries (USD/ha/yr) [11] |
|  | Distribution of fish or larvae [12] | Landings of commercial and recreational fishing (t, USD) [13] | Value of finfish (weighting factor) [4] |
|  | Fish biomass (standing stock) (t) [14] | Commercial fishery landings data [12] | Value of fish catch per household (USD/household/yr) [15] |
|  | Estimates of species abundance (fish, shellfish, marine mammals and birds) [16] | Fish catch per household (kg/household/yr) [15] | Net present value of shrimp and fish under different management scenarios (USD/ha) [17] |
|  | Proportion of fish stock overexploited, depleted or recovered (%) [18] | Fish catch (t) [19] | Shrimp revenues (USD/ha) [17] |
|  | Presence of reef-associated fish [20] | Fisheries production and non-marketed catch [21] | Annual value of fish catch (USD) [22] |
|  | Food web structure and robustness (various properties) [23] | Fish harvested by capture fisheries or produced in aquaculture [24] | Estimated value of fish catch per household (USD/ha/yr) [25] |
|  | Marine food chain [26] | Composition of local fisheries (harvest and catch size) [27] | Fisheries value (USD/km^2^)[6] |
|  | Presence of fry preys [28] | Predicted fish landings up to 2050 (t) [29] | Value of landed fish (GBP) [30] |
|  | Composition and relative importance of predators along a gradient of fishing intensities [31] | Fish production [32] | Predicted value of landings up to 2050 (USD/km^2^, Baht/km^2^) [29] |
|  | Functional variation of predatory performance (frequency of predation, ingestion time, urchin size selection) [31] | Landings (t) [33] | Annual net value (valuation method not specified) (CNY) [34] |
|  | Mangrove extent as habitat for fisheries (ha) [35] | Amount of fish from certified fisheries (t) [18] | Net value added: fish production from fisheries (EUR) [36] |
|  | State of the seagrass meadows [37] | Global landings from marine fisheries (t) [18] | Landings and landings value (t, USD) [38] |
|  | Diverging trends between area and productivity of mangrove forests [39] | Harvesting parameters [40] | Net value added: fish production from aquaculture (EUR)[36] |
|  | Area of marine protected areas (km^2^) [18] | Harvested fish and its consequences in the food web (USD/km^2^) [41] | Fish value (USD) [19] |
|  | Area of no take zones (km^2^) [18] | Fishery products (emergy exports from social-ecological systems) (J/yr) [42] | Economic value of fish for consumption and sale (SBD/household/ yr) [43] |
|  | Areas to support seafood production (ha) [44] | Spatial distribution of squid harvests (ranking) [45] | Market value of fish (KES) [46] |
|  | Carbon:nitrogen ratio [47] | Degree of specialization of fishing activities [39] | Net value of food production by mariculture (CNY) [48] |
|  | Primary production (gross, respiration and net) (mgC/m^2^/h) [47] | Marine farming [49] | Average annual earnings from fishing and fishing related activities (USD) [19] |
|  | Sea food productivity [49] | Reduction discard (%) [18] | Significance of fish sales for households (ranking)[43] |
|  | Sea food quality [49] | Depletion in the number of viable (non-collapsed) fisheries (%) [50] | Fish demand (Mt/yr) [29] |
|  | Fish food indicator [51] | Importance of mangroves for food (ranking)[52] | Total revenues from commercial and recreational fishing (USD) [53] |
|  |  | Spatial appropriation of marine ecosystems (ecological footprint) (m^2^) [54] | Market valuation for fisheries production [12] |
|  |  | Importance and specificity of food based on expert knowledge with reference to rabbits, asparagus, wild food, rare breed cattle, meat and miscellaneous crops in dunes (scores 0-3) [55] | Income from fisheries (USD/person/day) [56] |
|  |  |  | Local annual income linked to commercial fishing (EUR) [57] |
|  |  |  | Jobs linked to commercial fishing [57] |
|  |  |  | Employment in fisheries (n° employees) [18] |
|  |  |  | Employment linked to fishing and fishing related activities [19] |
|  |  |  | Social perception of fisheries (% respondents considering it important) [58] |
|  |  |  | Marginal value of a change in fisheries management [59] |
|  |  |  | Fishing income ratio (%) [39] |
|  |  |  | Propagule sales (SBD/household/yr) [43] |
|  |  |  | Change in the income from fishing and shrimping (%)[60] |
|  |  |  | Perceived benefit from commercial fishing, agriculture and livestock farming, bait digging (words used most frequently) [61] |
|  |  |  | Fishermen’s willingness-to-accept an alternative employment (MXN) [62] |
|  |  |  | Private and social benefits from different fishing activities under different conservation options (USD/km^2^) [17] |
|  |  |  | Records of illegal foreign fishing vessels (AUD) [63] |
|  |  |  | Monetary fisheries services by fringe mangroves (USD/ha)[64] |
|  |  |  | Value of mangroves use for aquaculture (USD/ha/yr) [65] |
|  |  |  | Value of total aquaculture sales [12] |
|  |  |  | Community perception on the importance of mangroves in food provision (honey and fisheries) (ranking) [66] |
|  |  |  | Coastal communities’ engagement in and reliance on fisheries [12] |
|  |  |  | Economic return of prawn, mollusk, shrimp and crab production (aquaculture) in mangroves (USD/yr) [67] |
|  |  |  | Benefits and costs of mangrove conversion for off-shore fisheries (USD/ha) [17] |
|  |  |  | Fleet capacity index (score 0-3) [18] |
|  |  |  | Economic losses of fishery industry caused by invasive species (EUR) [68] |
|  |  |  | Replacement cost with fishery restoration projects (USD/ha/yr) [69] |
|  |  |  | Crop productivity in coastal areas (land prices in USD) [70] |
| **Water storage and provision** |  | Importance and specificity of freshwater based on expert knowledge with reference to drinking water and irrigation (scores 0-3) [55] | Value of the water supply service based on benefit transfer (EUR/ha/yr) [71] |
|  |  | Importance and specificity based on expert knowledge with reference to drinking water and groundwater (scores 0-3) [55] | Value transfer based on Costanza et al. 1997 (USD) [72] |
|  |  | Importance and specificity of water storage based on expert knowledge (scores 0-3) [55] | Net value added: desalinated water supply (EUR) [36] |
|  |  |  | Replacement costs for alternative supply sources or water treatment cost savings [73] |
| **Biotic materials and biofuels** | Sponge diversity and abundance (weighting factor) [4] | Generation of sand and mangrove wood (weighting factor) [4] | Net present value of drugs from marine organisms (USD) [74] |
|  | Biomass production over stem diameter classes (tC/ha) [75] | Importance and specificity of fiber and fuel based on expert knowledge with reference to grass/reeds, wool and timber (scores 0-3) [55] | Finance including market value of raw materials (USD/ha/yr) [11] |
|  |  | Importance and specificity of mineral extraction based on expert knowledge with reference to sand and minerals (scores 0-3) [55] | Private and social benefits from coral mining under different conservation options (USD/km^2^) [17] |
|  |  | Importance and specificity of genetic resources based on expert knowledge with reference to breeding stock and biochemicals (scores 0-3) [55] | Market value of timber (KES) [46] |
|  |  | Importance of mangroves for wood (ranking) [52] | Net present value of clearance logging under different management scenarios (USD/ha) [17] |
|  |  | Importance of mangroves for construction (ranking) [52] | Net present value of fuelwood and timber under different management scenarios (USD/ha) [17] |
|  |  | Importance of mangroves for medicinal resources (ranking) [52] | Estimated value of wood for cooking (USD/ha/yr) [25] |
|  |  | Change in the use of mangroves as household fuel (%) [60] | Harvest and users of natural products [21] |
|  |  | Sand and gravel extraction (t) [76] | Quantity of wood collected and prices (SBD/household/yr) [43] |
|  |  | Household effort to collect firewood (h/week) [43] | Income related to seaweed farming (USD) [19] |
|  |  |  | Employment linked to seaweed farming [19] |
|  |  |  | Net value added of raw materials: seaweed, fishmeal, fish oil, ornamental (EUR) [36] |
|  |  |  | Total value of fishmeal and seaweeds (GBP) [30] |
|  |  |  | Social perception of energy provision (% respondents) [58] |
|  |  |  | Benefits and costs of mangrove conversion for timber and non-timber products (USD/ha) [17] |
|  |  |  | Annual revenues from mangrove resources (CFA franc per capita) [39] |
|  |  |  | Contribution of mangrove harvesting to GDP (USD) [19] |
|  |  |  | Community perception on the importance of mangroves in the provision of raw materials (ranking) [66] |
|  |  |  | Probability-based precautionary value of genetic resources based on expert judgment [73] |
| **Water purification** | Ammonium and phosphate concentration (microM) [77] | Oxygen concentration (mg/l) [77] | Cost of effluent treatment (CNY/kg) [48] |
|  | Particulate organic carbon (POC) and nitrogen (PON) (mg/l) [77] | Seston uptake or Chl-a removal (%) [78] | Replacement cost through sewage treatment (GBP) [79] |
|  | Suspended matter [80] | Nitrogen uptake (mmol N/m^3^/yr) [81] | Replacement cost of constructing a sewage treatment plant (USD/ha/yr) [21] |
|  | Bottom irradiance (micromol/m^2^/s) [77] | Quantity of nitrogen and phosphorus fixed by phytoplankton and kelp [48] | Significance of the toilet function for households (ranking) [43] |
|  | Presence of nitrophilous macroalgae in catchment basin [28] | Change in bioremediation capacity by algae and bivalves [82] | Contingent valuation of nitrogen reduction (SEK) [83] |
|  | Ecological risk indicator under different euthrophication scenarios [84] | Nitrogen removal rate (kgN/ha/yr) [5] | Cost of nutrient abatement (EUR/yr) [85] |
|  | Depletion in the number of suspension feeders, submerged vegetation and wetlands to filter water (%) [50] | Nitrogen and phosphorus retention (microg/l) [86] | Coastal recreation associated to reduce nutrient concentration (EUR/yr) [85] |
|  | Presence of floodplains, wetlands, estuaries, mangroves, benthic invertebrate species [13] | Nutrient abatement (t/yr) [85] | Benefit transfer valuation of water quality (USD/ha/yr) [11] |
|  | Number of dead zones [18] | Bacterial denitrification within the sediments [26] | Turnover rates and economic value of nitrogen, phosphorus and heavy metals concentration in sediments and biomass (USD/kg/yr) [65] |
|  | Plant tissue nitrogen concentration (%) [87] | Removal of total nutrient content (kg/ha) [88] | Net value added: replacement cost for the bioremediation of waste (EUR) [36] |
|  | Water circulation [89] | Nitrogen, phosphorus and heavy metals concentration and rate (kg, kg/yr) [65] | Value transfer based on Costanza et al. 1997 (USD ) [72] |
|  | Sedimentation and accumulation of organic matter [89] | Nitrogen accumulation (t/yr) [90] | Annual net value (valuation method not specified) (CNY) [34] |
|  | Ammonium and nitrate (mg ion/g resin) [87] | Denitrification (t/yr) [90] |  |
|  | Total soil nitrogen in a salt marsh (% dry weight) [87] | Oxygen levels in water and sediment [89] |  |
|  | Abundance of suspension and surface deposit feeder [89] | Particulate organic matter (POM) and photosynthetically active radiation (PAR) (mg AFDM/l, umol/m^2^/s)[47] |  |
|  | Presence of bioturbator organisms [28] | Chemical oxygen demand (COD) and biological oxygen demand (BOD) (mg/l) [91] |  |
|  | Nitrogen concentration (microM/l) [92] | Enhanced fishery catch through reduced eutrophication [73] |  |
|  | Presence of degrading microorganisms [28] | Spatial appropriation of marine ecosystems (ecological footprint) (m^2^) [54] |  |
|  | Distribution of *Phragmites Australis* [93] | Emergy flow accounting for environmental and economic inputs (solar emergy, sej/yr) [91] |  |
|  | Presence of suspension feeders [26] | Fecal colliform [80] |  |
|  | Feeding modes and impact on certain pollutants [26] |  |  |
|  | Seston reduction (mg/l) [8] |  |  |
| **Air quality regulation** |  | Importance and specificity of air quality regulation based on expert knowledge (scores 0-3) [55] |  |
| **Coastal protection** | Healthy growing coral reefs, mangroves and wetlands (%, USD) [13] | Surge reduction (cm/km) [5] | Value of the disturbance regulation service based on benefit transfer (EUR/ha/yr) [71] |
|  | Coral size and substrate cover [7] | Vulnerability index based on relaxation time and return interval [94] | Value transfer based on Costanza et al. 1997 (USD) [72] |
|  | Plant cover (%) [95] | Importance and specificity of storm protection based on expert knowledge (scores 0-3) [55] | Liberty index [96] |
|  | Vegetation properties (marsh width, species, biomass production, density, stiffness, height) [97] | Loss rates of experimental equipment in the coast (no. equipment lost) [98] | Gross Domestic Product [96] |
|  | Vegetation density (shoots/ha, g/m^2^, t/ha) [99] | Wave attenuation (m) [100] | Willingness-to-pay of local residents (GBP/yr) [79] |
|  | Temporal changes in mangrove extent (ha) [64] | Wave attenuation (m, %) [99] | Perceived benefit from coastal protection (words used most frequently) [61] |
|  | Mangrove extent (ha) [35] | Sediment deposition (%) [101] | Loss in property values from declining reef protection (USD/ha) [6] |
|  | Presence of seagrass meadow [28] |  | Loss in property values from declining shoreline protection (USD/ha) [6] |
|  | Kelp occurrence adjacent to human property (%) [45] |  | Replacement cost for coastal protection (USD/ha/yr) [11] |
|  | Coverage of semi-altered land use type (%) [96] |  | Maintenance cost of defences (GBP/km/yr) [102] |
|  | Hydrodynamics (hydroperiod, distance to a sediment supply) [97] |  | Maintenance cost of sea defences (GBP/km/yr) [103] |
|  | Aboveground biomass (g DW/ha) [99] |  | Preventative costs or money saved in sea defence construction (GBP/m^2^) [79] |
|  | Hurricane frequency [96] |  | Avoided cost value of shoreline protection (KES) [46] |
|  | Health of wetland ecosystem [73] |  | Avoidance cost based on the water-related post-tsunami damages in South Asia 2004 (USD/yr) [104] |
|  | Sediment accretion (mm) [105] |  | Replacement cost as the cost of constructing breakwaters (USD/yr) [104] |
|  | Change in erosion protection capacity [82] |  | Historical costs avoided for not having to replace engineered structures (USD/ha) [17] |
|  |  |  | Economic value of protection against natural disasters (USD/ha, USD/household) [21] |
|  |  |  | Net monetary value of storm protection (USD/ha) [6] |
|  |  |  | Significance of storm protection for households (ranking) [43] |
|  |  |  | Avoided storm-related deaths [106] |
|  |  |  | Mortality rate caused by cyclones, hurricanes and tropical storms in coastal zones [96] |
|  |  |  | Avoidance cost with respect to historical storms (USD/ha/yr) [69] |
|  |  |  | Avoided damage per storm condition and area of wetland (USD/ha/yr) [107] |
|  |  |  | Avoided cost of coastal disturbance (GBP) [30] |
|  |  |  | Moderating effects of wetlands on storm surges and avoided costs in property damage [73] |
|  |  |  | Net present value of wave attenuation (USD/km^2^) [99] |
|  |  |  | Economic estimate of protection by mangroves through damages to agriculture, livestock, fisheries, change in number of deaths (USD) [6] |
|  |  |  | Damage costs to aquatic infrastructure caused by invasive species [68] |
|  |  |  | Awareness on the protective capacity of mangroves against storms or erosion (% survey respondents) [25] |
|  |  |  | Awareness on the capacity of mangroves to protect the reefs (% survey respondents) [25] |
| **Climate regulation** | Standing carbon and nitrogen stock (mg/m^2^) [47] | Soil carbon accumulation (MgC/ha/yr) [5] | Carbon sequestration benefits (GBP/tC) [102] |
|  | Carbon and nitrogen concentration (g/m^2^) [101] | Carbon flow (TgC/yr) [108] | Quantity of carbon fixed combined with the marginal damage costs of carbon emissions (GBP) [79] |
|  | Carbon stock (t/ha) [109] | Net photosynthetic rate (kgC/ha/yr) [21] | Market value of carbon (KES) [46] |
|  | Estimates of the global pools of carbon and fluxes between them (Pg C, Pg C/yr) [110] | Primary production (gC/m^2^/yr) [81] | Value of carbon based on the Sweden carbon tax rate (CNY) [48] |
|  | Aboveground biomass and dissolved organic matter (gC/m^2^/yr) [111] | Carbon sequestration rate (gC/m^2^/yr) [112] | Net value added: primary production valued by the emission permits market (EUR) [36] |
|  | Dissolved organic and inorganic matter (gC/m^2^/yr)[111] | Oceanic uptake of carbon (Pg C/yr) [110] | Quantity of carbon sequestered by phytoplankton multiplied by the average price of carbon (GBP) [30] |
|  | Carbon biomass (t/ha) [113] | Microbial breakdown and deposit feeders activity in the sediments [26] | Net carbon production by plants multiplied by market cost to sequester carbon (USD/ha/yr) [69] |
|  | Carbon stock in the soil (kgC/ha) [21] | Leaf litter production (t DW/ha/yr) [100] | Price of traded carbon (GBP/t CO_2_e) [103] |
|  | Carbon fixed by phytoplankton, mariculture kelp and cultured shellfish (t) [48] | Importance and specificity of climate regulation based on expert knowledge (scores 0-3) [55] | Biomass production and its value (USD/ha/yr) [35] |
|  | Sediment carbon density (t/ha) [114] | Soil/sediment exchange of carbon monoxide, methane and nitrous oxide (microgC/m^2^/h) [114] | Annual net value (valuation method not specified) (CNY) [34] |
|  | Carbon sequestration potential (gC/yr) [112] |  |  |
|  | Carbon and nitrogen storage in canopies (kg/m^2^) [23] |  |  |
|  | Carbon cycling indicator [51] |  |  |
|  | Macrophyte biomass and carbon content (g/m^2^) [115] |  |  |
| **Ocean nourishment** | Nutrients stored in the sediments (mmol N/m^3^/yr) [81] | Importance and specificity of soil formation based on expert knowledge (scores 0-3) [55] | Value transfer based on Costanza et al. 1997 (USD) [72] |
|  | Nutrient transport to adjacent areas (mmol N/m^3^/yr) [81] | Importance and specificity of nutrient cycling based on expert knowledge (scores 0-3) [55] | Value of nutrient cycling in open ocean (GBP) [30] |
|  | Presence of four coralline algae [13] | Decomposition of dissolved and particulate organic matter by bacteria and funghi in the sediments [26] | Quantity of oxygen produced multiplied by the price of industrial oxygen (CNY) [48] |
|  | Nitrogen flux (mol N/yr) [116] | Oxygen emitted by primary production and kelp production (t) [48] |  |
|  | Environmental measurements: tidal inundation time (g/m^2^/h), net flux (g/m^2^/h), tidal height, salinity, nutrient concentrations (mg/l), nitrogen:phosphorus ratio [117] |  |  |
|  | Soil chemical properties (pH, organic carbon, total nitrogen, available phosphorus, potassium) (kg/ha) [70] |  |  |
|  | Nitrogen and phosphorus aboveground and in soil (g/m^2^) [115] |  |  |
|  | Silica fluxes (mol/h) [118] |  |  |
|  | Nutrient regeneration indicator [51] |  |  |
|  | Relationship between fish, bioturbation, bottom conditions and nutrients release [24] |  |  |
|  | Function of fish as active or passive transporters and distributors of energy and materials [24] |  |  |
| **Life cycle maintenance** | Substrate character [3] | Juvenile fish density (t) [119] | Value of the habitat service based on benefit transfer (EUR/ha/yr) [71] |
|  | Structural complexity, nursery and feeding areas [13] | Juvenile density (abundance/m^2^) [120] | Value transfer based on Costanza et al. 1997 (USD) [72] |
|  | Connectivity, diversity, trophic composition [13] | Postlarvae production per hatchery (no.postlarvae/yr) [22] | Annual value of ES based on benefit transfer (USD/yr) [121] |
|  | Total coral cover (m^2^) [122] | Effect of mangrove coverage on the total fishery value [123] | Tourism’s willingness-to-pay for reduced fishing pressure and coral reef conservation (USD) [62] |
|  | Composite metrics using percent cover of corals [122] | Annual production of fish juveniles (g/m^2^/yr) [124] | Contingent valuation of habitat service based on public preferential value (USD/ha/yr) [11] |
|  | Size-frequency distributions of corals [122] | Foraging efficiency for fish [89] | Regional GDP (t) [119] |
|  | Topographic complexity of corals [122] | Importance and specificity of the provision of habitat based on expert knowledge (scores 0-3) [55] | Habitat creation benefits (GBP/ha/yr) [102] |
|  | Coral extent and condition (km^2^) [18] | Importance and specificity of pollination in dunes based on expert knowledge (scores 0-3) [55] | Community perception on the importance of mangroves for habitat provision (living space for introduced and migratory species) (ranking) [66] |
|  | Diversity and abundance of cold-water corals [16] |  | Monetary returns of shrimp farming in mangroves (USD/ha) [6] |
|  | Nursery area (km^2^) [119] |  | Costs of mangrove rehabilitation (USD/ha) [6] |
|  | Eelgrass productivity (cm^2^/m^2^/d) [101] |  | Willingness-to-pay for the life-support input of shrimp spawners (USD/shrimp spawner) [22] |
|  | Natural size of mangroves and density progression [100] |  | Significance of nursery habitat for households (ranking) [43] |
|  | Mangrove and seagrass extent (km^2^) [18] |  | Social perception of existing conservation designations (% respondents considering it important) [58] |
|  | Mangrove biomass (t/yr) [90] |  | Price of the commercial fish species (GBP/kg/ha) [103] |
|  | Abundance of seagrasses (indiv/m^2^) [47] |  | Economic value of the annual juvenile fish production based on the price of aquaculture growth (JPY/g) [124] |
|  | Macrophyte species richness (no.species/m^2^) [115] |  |  |
|  | Distribution of *Phragmites Australis* [93] |  |  |
|  | Depletion in the number of oyster reefs, sea grass beds and wetlands to provide nursery (%)[50] |  |  |
|  | Protected area designated for its diversed habitat and abundant seabird colonies [76] |  |  |
|  | Habitat change (km^2^) [90] |  |  |
|  | Species abundance and richness (indiv/m^2^, spp/m^2^) [23] |  |  |
|  | Intertidal biodiversity [100] |  |  |
|  | Mechanical prevention of larval immigration [89] |  |  |
|  | Abundance of food organisms [89] |  |  |
|  | Consumption of organisms by fish/ foodchain relationships [24] |  |  |
|  | Biomass of sessile epifauna (g/m^2^) [125] |  |  |
|  | Oxygen level in water column [89] |  |  |
| **Biological regulation** |  | Control of aquatic disease bearing invertebrates and plants by fish [24] | Value transfer based on Costanza et al. 1997 (USD) [72] |
|  |  | Importance and specificity of pest regulation based on expert knowledge (scores 0-3) [55] |  |
|  |  | Importance and specificity of disease regulation based on expert knowledge (scores 0-3) [55] |  |
| **Symbolic and aesthetic values** |  | Number of landed individuals of ornamental invertebrates [126] | Value of the spiritual and historic service based on benefit transfer (EUR/ha/yr) [71] |
|  |  | Importance and specificity of aesthetic values based on expert knowledge (scores 0-3) [55] | Value transfer based on Costanza et al. 1997 (USD) [72] |
|  |  | Importance and specificity of cultural heritage based on expert knowledge (scores 0-3) [55] | Global aquarium industry [24] |
|  |  | Aesthetic enjoyment [49] | Price of a hotel room with sea views (EUR) [127] |
|  |  |  | Price of beach and non-beach plots of land (USD/ha) [9] |
|  |  |  | Respondents' preference and ranking (USD/ha) [9] |
|  |  |  | Hedonic pricing: cost of property next to aesthetic sites compared with those far away (USD/ha/yr) [11] |
|  |  |  | Hedonic pricing of aesthetic values of landscape change [73] |
|  |  |  | Willingness-to-accept compensation for cultural values (ranking) [35] |
|  |  |  | Public preference of seascape (%) [128] |
|  |  |  | Public preference of place identity (%)[128] |
|  |  |  | Social perception of identity/heritage (% respondents considering it important) [58] |
| **Recreation and tourism** | Size of marine leisure and recreation hotspots (km^2^) [129] | Beach closure due to bacteria limit, discolored or turbid water [80] | Value of the recreational and aesthetic service based on benefit transfer (EUR/ha/yr) [71] |
|  | Visual cover of algal mats [89] | Snorkeling and swimming (weighting factor) [4] | Willingness-to-pay for the enjoyment in the beach, before and after erosion (GBP) [130] |
|  | Smell of decomposing algae [89] | Importance and specificity of recreation and tourism based on expert knowledge (scores 0-3) [55] | Choice and willingness-to-pay by householders [131] |
|  | Physical disturbance on recreational activities [89] | Annual number of recreational trips (actual or intended) per person [132] | Value transfer based on Costanza et al. 1997 (USD) [72] |
|  | Presence of coralligenous community or cetacean population [28] | Primary contact recreation (bathing, diving) [49] | Annual value of ES based on benefit transfer (USD/yr) [121] |
|  |  | Importance of mangroves for tourism (ranking) [52] | Benefit transfer valuation for cultural services (USD/ha/yr) [11] |
|  |  | Whale/charismatic species watching [32] | Perceived benefit from walking, dog walking, cycling, running, wildlife watching, horse riding, golf, kite surfing and kite flying, windsurfing, kayaking, canoeing, metal detecting, wildfowling, angling, sailing and power boats, art and photography, camping (words used most frequently) [61] |
|  |  | Spatial distribution of whale boat visitation intensity (ranking) [45] | Perceived benefit from hotels, public houses and restaurants (words used most frequently) [61] |
|  |  | No. of tourists linked to sportive fishery [2] | Annual state-level estimates of economic value for beach-going, recreational fishing, marine bird life viewing, scuba diving and snorkeling [12] |
|  |  | No. of visitors and passengers on commercial tour boats (%, USD) [13] | Non-market value estimates for clean beaches, wildlife viewing, recreational fishing, and snorkeling [12] |
|  |  | Decrease in the number of tourists caused by invasive species (e.g. jellyfish) [68] | Beach visitors sensitivity to bacteria [80] |
|  |  |  | Beach visitors and travel cost [80] |
|  |  |  | No. of visitors and travel cost (USD/ha) [9] |
|  |  |  | Willingness-to-pay by tourists (USD/yr) [6] |
|  |  |  | Willingness-to-pay per person (USD/yr) [132] |
|  |  |  | Willingness-to-pay and consumer surplus per recreation benefit (USD/ha/yr) [69] |
|  |  |  | Tourist travel cost and public preference (USD/ha/yr) [11] |
|  |  |  | Market value of ecotourism (KES) [46] |
|  |  |  | Total income generated by tourism (USD) [19] |
|  |  |  | Total money associated to tourism emergy exports (USD) [42] |
|  |  |  | Employment linked to tourism [19] |
|  |  |  | Travel cost or contingent valuation [73] |
|  |  |  | Tourists’ perception in a MPA (awareness of being in a MPA, opinion on management activities, importance of natural attractiveness, satisfaction with recreational experience and willingness to come back, and awareness of environmental impacts) (% responses) [133] |
|  |  |  | Community perception on the importance of mangroves for tourism (ranking) [66] |
|  |  |  | Tourism potential value based on the environment (USD/ride) [65] |
|  |  |  | Social perception of tourism/leisure/recreation (% respondents) [58] |
|  |  |  | Jobs linked to recreational fishing [57] |
|  |  |  | Local annual income linked to recreational fishing (EUR) [57] |
|  |  |  | Value of sea anglers and divers activities (GBP) [129] |
|  |  |  | Visitor expenditure related to angling, diving and marine mammal watching (GBP) [30] |
|  |  |  | Marine mammal valuations for welfare and leisure [41] |
|  |  |  | Local annual income linked to scuba (EUR) [57] |
|  |  |  | Jobs linked to scuba [57] |
|  |  |  | Economic value of water quality based on a choice experiment (GBP/yr) [103] |
|  |  |  | Percentage of the GNP (USD) [20] |
|  |  |  | Recognition of customary use rights [21] |
| **Cognitive effects** | Fish studies as a source of information [24] | Curiosity (weighting factor) [4] | Perceived benefit from education, travelling community stop point, archaeology, housing, sense of well being and/or belonging (words used most frequently) [61] |
|  |  | Importance and specificity of education based on expert knowledge (scores 0-3) [55] | Value added by research and development, education and training (GBP) [30] |
|  |  | Importance and specificity of inspiration based on expert knowledge (scores 0-3) [55] | Research spending by NGOs (USD/ha) [9] |
|  |  |  | Financial expenditure in research (USD/ha/yr) [11] |
|  |  |  | Research funds value (KES) [46] |
|  |  |  | Significance of artistic raw materials for households (ranking) [43] |
|  |  |  | Net value added: Government financial transfers related to research, management, enforcement, MPA and opportunity cost (EUR) [36] |
|  |  |  | Social perception of cognitive value (% respondents considering it important) [58] |
| **All MCES together** | Hydrological regime affecting all ecosystem services [27] |  | Additional tax suggested by households to preserve marine environmental benefits (GBP/household/yr) [134] |
|  | Coverage of mangrove forests affecting all ecosystem services [27] |  | Implicit price of the willingness to conserve marine biodiversity (GBP/household/yr) [134] |
|  | Species richness [135] |  | Implicit price of the willingness to restrict marine activities and development (GBP/household/yr) [134] |
|  | Species diversity [136] |  | Total ecosystem service value for each land-cover category using benefit transfer from Costanza et al. (1997) (USD/yr)[137,138] |
|  | Species turnover [135] |  | Population density within 100km from the coastline (people/km^2^) [139] |
|  | Marine vertebrates living planet index (score 0-1.2) [18] |  | Ecosystem services product based on land cover, ecosystem equivalence and benefit transfer from Costanza et al. 1997 (USD/km^2^/ecosystem/yr) [139] |
|  | Pelagic seabird red list index (score 0.7-0.8) [18] |  | Global value of ES per biome based on Constanza et al. (1997) (USD/ha/yr) [140] |
|  | Local extinctions [135] |  | Contingent valuation of biodiversity based on questionnaires and financial expenditure (USD/ha/yr) [11] |
|  | Invasions intensity [135] |  | Trend and importance in ecosystem services provision as perceived by local stakeholders (%) [27] |
|  | Extent of terrestrial and marine ecosystems (%) [141] |  | Net value added: Real Option Value of biomass conservation from financial economy (EUR) [36] |
|  | Importance of mangroves for biodiversity (ranking) [52] |  | Social perception of regulation services (% respondents considering it important) [58] |
|  | Ecosystem natural state [32] |  | Social perception of ecological value (% respondents considering it important) [58] |
|  | Habitat loss and degradation [142] |  | Social perception of supporting services (% respondents considering it important) [58] |
|  | Species abundance and community structure [142] |  | Social perception of all ecosystem services (% respondents considering it important) [58] |
|  | Shifts in the distribution of species and biomes [142] |  | Well-being indicator (score) [141] |
|  | Species extinctions [142] |  | Economic indicator (score) [141] |
|  |  |  | Valuation index for a policy problem based on stakeholders' opinion [143] |
|  |  |  | Multi-attribute utility theory index of ES (natural units) [143] |
|  |  |  | Explicit consideration of the beneficiaries of ES [144] |
|  |  |  | Mechanisms of access of individuals to ES [144] |
|  |  |  | Well-being context: the perspectives and circumstances of ES beneficiaries that contribute to well-being [144] |
|  |  |  | Equity weights [144] |
|  |  |  | Cash income and employment [144] |

List of indicators and units found during the systematic review of published marine and coastal ecosystem service (MCES) assessments. Indicators are classified following the cascade model into capacity, flow and benefit (see section 2.4 and fig. 2).

References:

1. Brehmer P, Do Chi T, Laugier T, Galgani F, Laloë F, et al. (2011) Field investigations and multi-indicators for shallow water lagoon management: Perspective for societal benefit. Aquatic Conservation: Marine and Freshwater Ecosystems 21: 728–742.

2. Leslie HM, Schlüter M, Cudney-Bueno R, Levin SA (2009) Modeling responses of coupled social-ecological systems of the Gulf of California to anthropogenic and natural perturbations. Ecological Research 24: 505–519.

3. McAdoo BG, Ah-Leong JS, Bell L, Ifopo P, Ward J, et al. (2011) Coral reefs as buffers during the 2009 South Pacific tsunami, Upolu Island, Samoa. Earth-Science Reviews 107: 147–155.

4. Mumby PJ, Broad K, Brumbaugh DR, Dahlgren CP, Harborne AR, et al. (2008) Coral reef habitats as surrogates of species, ecological functions, and ecosystem services. Conservation Biology 22: 941–951.

5. Engle VD (2011) Estimating the provision of ecosystem services by Gulf of Mexico coastal wetlands. Wetlands 31: 179–193.

6. Barbier EB, Hacker SD, Kennedy C, Koch EW, Stier AC, et al. (2011) The value of estuarine and coastal ecosystem services. Ecological Monographs 81: 169–193.

7. Harris A, Manahira G, Sheppard A, Gough C, Sheppard C (2010) Demise of Madagascar’s once great barrier reef-change in coral reef condition over 40 years. Atoll Research Bulletin: 1–16.

8. North EW, King DM, Xu J, Hood R, Newell RIE, et al. (2010) Linking optimization and ecological models in a decision support tool for oyster restoration and management. Ecological Applications 20: 851–866.

9. Hicks CC, McClanahan TR, Cinner JE, Hills JM (2009) Trade-offs in values assigned to ecological goods and services associated with different coral reef management strategies. Ecology and Society 14: 0.

10. Wilson SK, Fisher R, Pratchett MS, Graham NAJ, Dulvy NK, et al. (2008) Exploitation and habitat degradation as agents of change within coral reef fish communities. Global Change Biology 14: 2796–2809.

11. Hicks CC (2011) How do we value our reefs? Risks and tradeoffs across scales in “biomass-based” economies. Coastal Management 39: 358–376. doi:10.1080/08920753.2011.589219.

12. Lester SE, McLeod KL, Tallis H, Ruckelshaus M, Halpern BS, et al. (2010) Science in support of ecosystem-based management for the US West Coast and beyond. Biological Conservation 143: 576–587.

13. Stoeckl N, Hicks CC, Mills M, Fabricius K, Esparon M, et al. (2011) The economic value of ecosystem services in the Great Barrier Reef: Our state of knowledge. Annals of the New York Academy of Sciences 1219: 113–133.

14. Edwards HJ, Elliott IA, Pressey RL, Mumby PJ (2010) Incorporating ontogenetic dispersal, ecological processes and conservation zoning into reserve design. Biological Conservation 143: 457–470.

15. Hussain SA, Badola R (2010) Valuing mangrove benefits: Contribution of mangrove forests to local livelihoods in Bhitarkanika Conservation Area, East Coast of India. Wetlands Ecology and Management 18: 321–331.

16. Schumacher JD, Kruse GH (2005) Toward sustainable ecosystem services from the Aleutian Archipelago. Fisheries Oceanography 14: 277–291.

17. Turner RK, Paavola J, Cooper P, Farber S, Jessamy V, et al. (2003) Valuing nature: Lessons learned and future research directions. Ecological Economics 46: 493–510.

18. Sparks TH, Butchart SHM, Balmford A, Bennun L, Stanwell-Smith D, et al. (2011) Linked indicator sets for addressing biodiversity loss. ORYX 45: 411–419.

19. Lange G-M, Jiddawi N (2009) Economic value of marine ecosystem services in Zanzibar: Implications for marine conservation and sustainable development. Ocean & Coastal Management 52: 521–532.

20. Souter DW, Lindén O (2000) The health and future of coral reef systems. Ocean and Coastal Management 43: 657–688.

21. Walters BB, Rönnbäck P, Kovacs JM, Crona B, Hussain SA, et al. (2008) Ethnobiology, socio-economics and management of mangrove forests: A review. Aquatic Botany 89: 220–236.

22. Rönnbäck P, Troell M, Zetterström T, Babu DE (2003) Mangrove dependence and socio-economic concerns in shrimp hatcheries of Andhra Pradesh, India. Environmental Conservation 30: 344–352.

23. Schmidt AL, Coll M, Romanuk TN, Lotze HK (2011) Ecosystem structure and services in eelgrass Zostera marina and rockweed Ascophyllumnodosum habitats. Marine Ecology Progress Series 437: 51–68.

24. Holmlund CM, Hammer M (1999) Ecosystem services generated by fish populations. Ecological Economics 29: 253–268.

25. Naylor R, Drew M (1998) Valuing mangrove resources in Kosrae, Micronesia. Environment and Development Economics 3: 471–490.

26. Snelgrove PVR, Henry Blackburn T, Hutchings PA, Alongi DM, Frederick Grassle J, et al. (1997) The importance of marine sediment biodiversity in ecosystem processes. Ambio 26: 578–583.

27. Vilardy SP, González JA, Martín-López B, Montes C (2011) Relationships between hydrological regime and ecosystem services supply in a Caribbean coastal wetland: A social-ecological approach. Relations entre reacute;gime hydrologique et fourniture de services eacute;cosysteacute;miques dans une zonehumide côtière des Caraïbes: Une approche socio-écologique 56: 1423–1435.

28. Cognetti G, Maltagliati F (2010) Ecosystem service provision: an operational way for marine biodiversity conservation and management. Marine pollution bulletin 60: 1916–1923.

29. Alcamo J, Van Vuuren D, Ringler C, Cramer W, Masui T, et al. (2005) Changes in nature’s balance sheet: Model-based estimates of future worldwide ecosystem services. Ecology and Society 10.

30. Hussain SS, Winrow-Giffin A, Moran D, Robinson LA, Fofana A, et al. (2010) An ex ante ecological economic assessment of the benefits arising from marine protected areas designation in the UK. Ecological Economics 69: 828–838.

31. Clemente S, Hernández JC, Rodríguez A, Brito A (2010) Identifying keystone predators and the importance of preserving functional diversity in sublittoral rocky-bottom areas. Marine Ecology Progress Series 413: 55–67.

32. Eichner T, Tschirhart J (2007) Efficient ecosystem services and naturalness in an ecological/economic model. Environmental and Resource Economics 37: 733–755.

33. Limburg KE, Waldman JR (2009) Dramatic declines in north Atlantic diadromous fishes. BioScience 59: 955–965.

34. Shi H.a b Zheng W. WZ. DD., Shi H, Zheng W, Wang Z, Ding D (2009) Sensitivity and uncertainty analysis of regional marine ecosystem services value. Journal of Ocean University of China 8: 150–154.

35. Martinez-Alier J (2001) Ecological conflicts and valuation: Mangroves versus shrimps in the late 1990s. Environment and Planning C: Government and Policy 19: 713–728.

36. Murillas-Maza A, Virto J, Gallastegui MC, González P, Fernández-Macho J (2011) The value of open ocean ecosystems: A case study for the Spanish exclusive economic zone. Natural Resources Forum 35: 122–133.

37. Unsworth RK, Cullen LC (2010) Recognising the necessity for Indo-Pacific seagrass conservation. Conservation Letters 3: 63–73.

38. Hunsicker ME, Essington TE, Watson R, Sumaila UR (2010) The contribution of cephalopods to global marine fisheries: can we have our squid and eat them too? Fish and Fisheries 11: 421–438.

39. Conchedda G, Lambin EF, Mayaux P (2011) Between land and sea: Livelihoods and environmental changes in mangrove ecosystems of Senegal. Annals of the Association of American Geographers 101: 1259–1284.

40. Finnoff D, Gong M, Tschirhart J (2012) Perspectives on Ecosystem Based Management for Delivering Ecosystem Services with an Example from an Eighteen-Species Marine Model. International Review of Environmental and Resource Economics 6: 79–118.

41. Finnoff D, Tschirhart J (2008) Linking dynamic economic and ecological general equilibrium models. Resource and Energy Economics 30: 91–114.

42. Lomas PL, Álvarez S, Rodríguez M, Montes C (2008) Environmental accounting as a management tool in the Mediterranean context: The Spanish economy during the last 20 years. Journal of Environmental Management 88: 326–347.

43. Warren-Rhodes K, Schwarz A-M, Boyle LN, Albert J, Agalo SS, et al. (2011) Mangrove ecosystem services and the potential for carbon revenue programmes in Solomon Islands. Environmental Conservation 38: 485–496.

44. Folke C, Kautsky N, Berg H, Jansson Å, Troell M (1998) The ecological footprint concept for sustainable seafood production: A review. Ecological Applications 8.

45. Tallis H, Lester SE, Ruckelshaus M, Plummer M, McLeod K, et al. (2012) New metrics for managing and sustaining the ocean’s bounty. Marine Policy 36: 303–306.

46. Kairo JG, Wanjiru C, Ochiewo J (2009) Net pay: Economic analysis of a replanted mangrove plantation in Kenya. Journal of Sustainable Forestry 28: 395–414.

47. Antón A, Cebrian J, Heck KL, Duarte CM, Sheehan KL, et al. (2011) Decoupled effects (positive to negative) of nutrient enrichment on ecosystem services. Ecological Applications 21: 991–1009.

48. Zheng W, Shi H, Chen S, Zhu M, Zheng W.a b Shi H. b CS. ZM. (2009) Benefit and cost analysis of mariculture based on ecosystem services. Ecological Economics 68: 1626–1632.

49. Xu FL, Lam KC, Zhao ZY, Zhan W, Chen YD, et al. (2004) Marine coastal ecosystem health assessment: A case study of the Tolo Harbour, Hong Kong, China. Ecological Modelling 173: 355–370.

50. Worm B, Barbier EB, Beaumont N, Duffy JE, Folke C, et al. (2006) Impacts of biodiversity loss on ocean ecosystem services. Science 314: 787–790.

51. Frid CLJ (2011) Temporal variability in the benthos: Does the sea floor function differently over time? Journal of Experimental Marine Biology and Ecology 400: 99–107.

52. López-Medellín X, Castillo A, Ezcurra E (2011) Contrasting perspectives on mangroves in arid Northwestern Mexico: Implications for integrated coastal management. Ocean and Coastal Management 54: 318–329.

53. Cooley SR, Doney SC (2009) Anticipating ocean acidification’s economic consequences for commercial fisheries. Environmental Research Letters 4: 0.

54. Jansson A, Folke C, Rockström J, Gordon L (1999) Linking freshwater flows and ecosystem services appropriated by people: The case of the Baltic Sea drainage basin. Ecosystems 2: 351–366.

55. Everard M, Jones L, Watts B (2010) Have we neglected the societal importance of sand dunes? An ecosystem services perspective. Aquatic Conservation: Marine and Freshwater Ecosystems 20: 476–487.

56. Kronen M, Vunisea A, Magron F, McArdle B (2010) Socio-economic drivers and indicators for artisanal coastal fisheries in Pacific island countries and territories and their use for fisheries management strategies. Marine Policy 34: 1135–1143.

57. Roncin N, Alban F, Charbonnel E, Crec’hriou R, De la Cruz Modino R, et al. (2008) Uses of ecosystem services provided by MPAs: How much do they impact the local economy? A southern Europe perspective. Journal for Nature Conservation 16: 256–270.

58. Ruiz-Frau A, Edwards-Jones G, Kaiser MJ (2011) Mapping stakeholder values for coastal zone management. Marine Ecology Progress Series 434: 239–249.

59. Mäler K-G, Aniyar S, Jansson Å (2009) Accounting for ecosystems. Environmental and Resource Economics 42: 39–51.

60. McNally CG, Uchida E, Gold AJ (2011) The effect of a protected area on the tradeoffs between short-run and long-run benefits from mangrove ecosystems. Proceedings of the National Academy of Sciences of the United States of America 108: 13945–13950.

61. Holt AR, Godbold JA, White PCL, Slater A-M, Pereira EG, et al. (2011) Mismatches between legislative frameworks and benefits restrict the implementation of the Ecosystem Approach in coastal environments. Marine Ecology Progress Series 434: 213–228.

62. Barr RF, Mourato S (2009) Investigating the potential for marine resource protection through environmental service markets: An exploratory study from La Paz, Mexico. Ocean and Coastal Management 52: 568–577.

63. Muller S (2008) Indigenous Payment for Environmental Service (PES) Opportunities in the Northern Territory: Negotiating with customs. Australian Geographer 39: 149–170.

64. López-Medellín X, Ezcurra E, González-Abraham C, Hak J, Santiago LS, et al. (2011) Oceanographic anomalies and sea-level rise drive mangroves inland in the Pacific coast of Mexico. Journal of Vegetation Science 22: 143–151.

65. Souza FES, Ramos e Silva CA (2011) Ecological and economic valuation of the Potengi estuary mangrove wetlands (NE, Brazil) using ancillary spatial data. Journal of Coastal Conservation 15: 195–206.

66. Iftekhar MS, Takama T (2008) Perceptions of biodiversity, environmental services, and conservation of planted mangroves: A case study on Nijhum Dwip Island, Bangladesh. Wetlands Ecology and Management 16: 119–137.

67. Ellison AM (2008) Managing mangroves with benthic biodiversity in mind: Moving beyond roving banditry. Journal of Sea Research 59: 2–15.

68. Vilà M, Basnou C, Pyšek P, Josefsson M, Genovesi P, et al. (2010) How well do we understand the impacts of alien species on ecosystem services? A pan-European, cross-taxa assessment. Frontiers in Ecology and the Environment 8: 135–144.

69. Feagin RA, Martinez ML, Mendoza-Gonzalez G, Costanza R (2010) Salt marsh zonal migration and ecosystem service change in response to global sea level rise: A case study from an urban region. Ecology and Society 15.

70. Hussain SA, Badola R (2008) Valuing mangrove ecosystem services: Linking nutrient retention function of mangrove forests to enhanced agroecosystem production. Wetlands Ecology and Management 16: 441–450.

71. Lozoya JP, Sardá R, Jiménez JA (2011) A methodological framework for multi-hazard risk assessment in beaches. Environmental Science and Policy 14: 685–696.

72. Brenner J, Jiménez JA, Sardá R, Garola A (2010) An assessment of the non-market value of the ecosystem services provided by the Catalan coastal zone, Spain. Ocean and Coastal Management 53: 27–38.

73. Farber S, Costanza R, Childers DL, Erickson J, Gross K, et al. (2006) Linking ecology and economics for ecosystem management. BioScience 56: 121–133.

74. Erwin P, López-Legentil S, Schuhmann PW (2010) The pharmaceutical value of marine biodiversity for anti-cancer drug discovery. Ecological Economics 70: 445–451.

75. Fontalvo-Herazo ML, Piou C, Vogt J, Saint-Paul U, Berger U (2011) Simulating harvesting scenarios towards the sustainable use of mangrove forest plantations. Wetlands Ecology and Management 19: 397–407.

76. Atkins JP, Burdon D, Elliott M, Gregory AJ (2011) Management of the marine environment: integrating ecosystem services and societal benefits with the DPSIR framework in a systems approach. Marine Pollution Bulletin 62: 215–526.

77. Plutchak R, Major K, Cebrian J, Foster CD, Miller M-EC, et al. (2010) Impacts of Oyster Reef Restoration on Primary Productivity and Nutrient Dynamics in Tidal Creeks of the North Central Gulf of Mexico. Estuaries and Coasts 33: 1355–1364.

78. Grizzle RE, Greene JK, Coen LD (2008) Seston removal by natural and constructed intertidal eastern oyster (Crassostrea virginica) reefs: A comparison with previous laboratory studies, and the value of in situ methods. Estuaries and Coasts 31: 1208–1220.

79. Mangi SC, Davis CE, Payne LA, Austen MC, Simmonds D, et al. (2011) Valuing the regulatory services provided by marine ecosystems. Environmetrics 22: 686–698.

80. Tomlinson B, Sastre S, Blasco D, Guillén J (2011) The systems approach framework as a complementary methodology of adaptive management: A case study in the urban beaches of Barcelona. Ecology and Society 16.

81. Nunneri C, Lenhart HJ, Burkhard B, Windhorst W (2008) Ecological risk as a tool for evaluating the effects of offshore wind farm construction in the North Sea. Regional Environmental Change 8: 31–43.

82. Wallentinus I, Nyberg CD (2007) Introduced marine organisms as habitat modifiers. Marine Pollution Bulletin 55: 323–332.

83. Hökby S, Söderqvist T (2003) Elasticities of Demand and Willingness to Pay for Environmental Services in Sweden. Environmental and Resource Economics 26: 361–383.

84. Nunneri C, Windhorst W, Kerry Turner R, Lenhart H (2007) Nutrient emission reduction scenarios in the North Sea: An abatement cost and ecosystem integrity analysis. Ecological Indicators 7: 776–792.

85. Hyytiainen K, Ahtiainen H, Heikkilä J, Helin J, Huhtala A, et al. (2009) An integrated simulation model to evaluate national measures for the abatement of agricultural nutrients in the Baltic Sea. Agricultural and Food Science 18: 440–459.

86. Hansson L-A, Brönmark C, Nilsson PA, Åbjörnsson K (2005) Conflicting demands on wetland ecosystem services: Nutrient retention, biodiversity or both? Freshwater Biology 50: 705–714.

87. Fitch R, Theodose T, Dionne M (2009) Relationships among upland development, nitrogen, and plant community composition in a Maine Salt marsh. Wetlands 29: 1179–1188.

88. Higgins CB, Stephenson K, Brown BL (2011) Nutrient bioassimilation capacity of aquacultured oysters: Quantification of an ecosystem service. Journal of Environmental Quality 40: 271–277.

89. Troell M, Pihl L, Rönnbäck P, Wennhage H, Söderqvist T, et al. (2005) Regime shifts and ecosystem services in Swedish coastal soft bottom habitats: When resilience is undesirable. Ecology and Society 10.

90. Craft C, Clough J, Ehman J, Jove S, Park R, et al. (2009) Forecasting the effects of accelerated sea-level rise on tidal marsh ecosystem services. Frontiers in Ecology and the Environment 7: 73–78.

91. Vassallo P, Paoli C, Fabiano M (2009) Emergy required for the complete treatment of municipal wastewater. Ecological Engineering 35: 687–694.

92. Voss M, Dippner JW, Humborg C, Hürdler J, Korth F, et al. (2011) History and scenarios of future development of Baltic Sea eutrophication. Estuarine, Coastal and Shelf Science 92: 307–322.

93. Hershner C, Havens KJ (2008) Managing invasive aquatic plants in a changing system: Strategic consideration of ecosystem services. Conservation Biology 22: 544–550.

94. Pethick JS, Crooks S (2000) Development of a coastal vulnerability index: A geomorphological perspective. Environmental Conservation 27: 359–367.

95. Moreno-Casasola P, Martínez ML, Castillo-Campos G (2008) Designing ecosystems in degraded tropical coastal dunes. Ecoscience 15: 44–52.

96. Pérez-Maqueo O, Intralawan A, Martínez ML (2007) Coastal disasters from the perspective of ecological economics. Ecological Economics 63: 273–284.

97. Shepard CC, Crain CM, Beck MW (2011) The protective role of coastal marshes: A systematic review and meta-analysis. PLoS ONE 6.

98. Granek EF, Ruttenberg BI (2007) Protective capacity of mangroves during tropical storms: A case study from “Wilma” and “Gamma” in Belize. Marine Ecology Progress Series 343: 101–105.

99. Koch EW, Barbier EB, Silliman BR, Reed DJ, Perillo GME, et al. (2009) Non-linearity in ecosystem services: Temporal and spatial variability in coastal protection. Frontiers in Ecology and the Environment 7: 29–37.

100. Iftekhar MS (2008) Functions and development of reforested mangrove areas: A review. International Journal of Biodiversity Science and Management 4: 1–14.

101. McGlathery KJ, Reynolds LK, Cole LW, Orth RJ, Marion SR, et al. (2012) Recovery trajectories during state change from bare sediment to eelgrass dominance. Marine Ecology Progress Series 448: 209–221.

102. Turner RK, Burgess D, Hadley D, Coombes E, Jackson N (2007) A cost-benefit appraisal of coastal managed realignment policy. Global Environmental Change 17: 397–407.

103. Luisetti T, Turner K, Bateman I (2008) An ecosystem services approach to assess managed realignment coastal policy in England. Working Paper - Centre for Social and Economic Research on the Global Environment: 25.

104. Sanford MP (2009) Valuating mangrove ecosystems as coastal protection in post-tsunami South Asia. Natural Areas Journal 29: 91–95.

105. Bos AR, Bouma TJ, De Kort GLJ, Van Katwijk MM (2007) Ecosystem engineering by annual intertidal seagrass beds: Sediment accretion and modification. Estuarine, Coastal and Shelf Science 74: 344–348.

106. Das S, Vincent JR (2009) Mangroves protected villages and reduced death toll during Indian super cyclone. Proceedings of the National Academy of Sciences of the United States of America 106: 7357–7360.

107. Costanza R, Pérez-Maqueo O, Martinez ML, Sutton P, Anderson SJ, et al. (2008) The value of coastal wetlands for hurricane protection. Ambio 37: 241–248.

108. Alongi DM (2011) Carbon payments for mangrove conservation: Ecosystem constraints and uncertainties of sequestration potential. Environmental Science and Policy 14: 462–470.

109. Kauffman JB, Heider C, Cole TG, Dwire KA, Donato DC (2011) Ecosystem carbon stocks of micronesian mangrove forests. Wetlands 31: 343–352.

110. Lal R (2008) Sequestration of atmospheric CO2 in global carbon pools. Energy and Environmental Science 1: 86–100.

111. Lee SY (2008) Mangrove macrobenthos: Assemblages, services, and linkages. Journal of Sea Research 59: 16–29.

112. Xiaonan D, Xiaoke W, Lu F, Zhiyun O (2008) Primary evaluation of carbon sequestration potential of wetlands in China. Acta Ecologica Sinica 28: 463–469.

113. Donato DC, Kauffman JB, Murdiyarso D, Kurnianto S, Stidham M, et al. (2011) Mangroves among the most carbon-rich forests in the tropics. Nature Geoscience 4: 293–297.

114. Livesley SJ, Andrusiak SM (2012) Temperate mangrove and salt marsh sediments are a small methane and nitrous oxide source but important carbon store. Estuarine, Coastal and Shelf Science 97: 19–27.

115. Wieski K, Guo H, Craft CB, Pennings SC (2010) Ecosystem functions of tidal fresh, brackish, and salt marshes on the Georgia coast. Estuaries and Coasts 33: 161–169.

116. Roman J, McCarthy JJ (2010) The whale pump: Marine mammals enhance primary productivity in a coastal basin. PLoS ONE 5.

117. Adame MF, Virdis B, Lovelock CE (2010) Effect of geomorphological setting and rainfall on nutrient exchange in mangroves during tidal inundation. Marine and Freshwater Research 61: 1197–1206.

118. Vieillard AM, Fulweiler RW, Hughes ZJ, Carey JC (2011) The ebb and flood of Silica: Quantifying dissolved and biogenic silica fluxes from a temperate salt marsh. Estuarine, Coastal and Shelf Science 95: 415–423.

119. Cordier M, Pérez Agúndez JA, O’Connor M, Rochette S, Hecq W (2011) Quantification of interdependencies between economic systems and ecosystem services: An input-output model applied to the Seine estuary. Ecological Economics 70: 1660–1671.

120. Kamenos NA, Moore PG, Hall-Spencer JM (2004) Small-scale distribution of juvenile gadoids in shallow inshore waters; what role does maerl play? ICES Journal of Marine Science 61: 422–429.

121. McGlashan DJ, Firn JR (2003) Perceived socio-economic and environmental costs and benefits of ICZM in Scotland. Scottish Geographical Journal 119: 103–119.

122. Viehman S, Thur SM, Piniak GA (2009) Coral reef metrics and habitat equivalency analysis. Ocean and Coastal Management 52: 181–188.

123. Sanchirico JN, Mumby P (2009) Mapping ecosystem functions to the valuation of ecosystem services: Implications of species-habitat associations for coastal land-use decisions. Theoretical Ecology 2: 67–77.

124. Kamimura Y, Kasai A, Shoji J (2011) Production and prey source of juvenile black rockfish Sebastes cheni in a seagrass and macroalgal bed in the Seto Inland Sea, Japan: Estimation of the economic value of a nursery. Aquatic Ecology 45: 367–376.

125. Lambert GI, Jennings S, Kaiser MJ, Hinz H, Hiddink JG (2011) Quantification and prediction of the impact of fishing on epifaunal communities. Marine Ecology Progress Series 430: 71–86.

126. Rhyne A, Rotjan R, Bruckner A, Tlusty M (2009) Crawling to collapse: Ecologically unsound ornamental invertebrate fisheries. PLoS ONE 4.

127. Fleischer A (2012) A room with a view-A valuation of the Mediterranean Sea view. Tourism Management 33: 598–602.

128. Gee K, Burkhard B, Gee K.a Burkhard B. (2010) Cultural ecosystem services in the context of offshore wind farming: A case study from the west coast of Schleswig-Holstein. Ecological Complexity 7: 349–358.

129. Rees SE, Rodwell LD, Attrill MJ, Austen MC, Mangi SC (2010) The value of marine biodiversity to the leisure and recreation industry and its application to marine spatial planning. Marine Policy 34: 868–875.

130. Polyzos S, Minetos D (2007) Valuing environmental resources in the context of flood and coastal defence project appraisal: A case-study of Poole Borough Council seafront in the UK. Management of Environmental Quality 18: 684–710.

131. Christie M, Gibbons J (2011) The effect of individual “ability to choose” (scale heterogeneity) on the valuation of environmental goods. Ecological Economics 70: 2250–2257.

132. Bergstrom JC, Dorfman JH, Loomis JB (2004) Estuary management and recreational fishing benefits. Coastal Management 32: 417–432.

133. Petrosillo I, Zurlini G, Corlianò ME, Zaccarelli N, Dadamo M (2007) Tourist perception of recreational environment and management in a marine protected area. Landscape and Urban Planning 79: 29–37.

134. McVittie A, Moran D (2010) Valuing the non-use benefits of marine conservation zones: An application to the UK Marine Bill. Ecological Economics 70: 413–424.

135. Cheung WWL, Lam VWY, Sarmiento JL, Kearney K, Watson R, et al. (2009) Projecting global marine biodiversity impacts under climate change scenarios. Fish and Fisheries 10: 235–251.

136. Duarte CM (2000) Marine biodiversity and ecosystem services: An elusive link. Journal of Experimental Marine Biology and Ecology 250: 117–131.

137. Zhao B, Kreuter U, Li B, Ma Z, Chen J, et al. (2004) An ecosystem service value assessment of land-use change on Chongming Island, China. Land Use Policy 21: 139–148.

138. Zhao B, Li B, Zhong Y, Nakagoshi N, Chen J-K (2005) Estimation of ecological service values of wetlands in Shanghai, China. Chinese Geographical Science 15: 151–156.

139. Martínez ML, Intralawan A, Vázquez G, Pérez-Maqueo O, Sutton P, et al. (2007) The coasts of our world: Ecological, economic and social importance. Ecological Economics 63: 254–272.

140. Johnston P, Santillo D, Stringer R (1999) Marine environmental protection, sustainability and the precautionary principle. Natural Resources Forum 23: 157–167.

141. Bohensky E, Butler JRA, Costanza R, Bohnet I, Delisle A, et al. (2011) Future makers or future takers? A scenario analysis of climate change and the Great Barrier Reef. Global Environmental Change 21: 876–893.

142. Pereira HM, Leadley PW, Proença V, Alkemade R, Scharlemann JPW, et al. (2010) Scenarios for global biodiversity in the 21st century. Science 330: 1496–1501.

143. Carriger JF, Barron MG (2011) Minimizing risks from spilled oil to ecosystem services using influence diagrams: The Deepwater Horizon spill response. Environmental Science and Technology 45: 7631–7639.

144. Daw T, Brown K, Rosendo S, Pomeroy R (2011) Applying the ecosystem services concept to poverty alleviation: The need to disaggregate human well-being. Environmental Conservation 38: 370–379.
